# X-Ray Detected Magnetic Resonance: A Unique Probe of the Precession Dynamics of *Orbital* Magnetization Components

**DOI:** 10.3390/ijms12128797

**Published:** 2011-12-02

**Authors:** Jośe Goulon, Andrei Rogalev, Gérard Goujon, Fabrice Wilhelm, Jamal Ben Youssef, Claude Gros, Jean-Michel Barbe, Roger Guilard

**Affiliations:** 1 European Synchrotron Radiation Facility, B.P. 220, F-38043 Grenoble Cedex, France; 2 Laboratoire de Magńetisme de Bretagne, CNRS FRE 2697, UFR Sciences et Techniques, F-29328 Brest Cedex, France; 3 Institut de Chimie Moĺeculaire de l’Université de Bourgogne, UMR CNRS 5260, Groupe LIMRES, 9 Avenue Alain Savary, BP 47870, F-21078 Dijon Cedex, France

**Keywords:** XDMR, XMCD, FMR, high field EPR, AFMR

## Abstract

X-ray Detected Magnetic Resonance (XDMR) is a novel spectroscopy in which X-ray Magnetic Circular Dichroism (XMCD) is used to probe the resonant precession of *local* magnetization components in a strong microwave pump field. We review the conceptual bases of XDMR and recast them in the general framework of the linear and nonlinear theories of ferromagnetic resonance (FMR). Emphasis is laid on the information content of XDMR spectra which offer a unique opportunity to disentangle the precession dynamics of spin and orbital magnetization components at given absorbing sites. For the sake of illustration, we focus on selected examples in which marked differences were found between FMR and XDMR spectra simultaneously recorded on ferrimagnetically ordered iron garnets. With pumping capabilities extended up to sub-THz frequencies, high-field XDMR should allow us to probe the precession of orbital magnetization components in paramagnetic organometallic complexes with large zero-field splitting. Even more challenging, we suggest that XDMR spectra might be recorded on selected antiferromagnetic crystals for which orbital magnetism is most often ignored in the absence of any supporting experimental evidence.

## 1. Introduction

Orbital magnetism refers to a subtle interplay among several effects: Coulomb and spin-orbit interactions, hybridization and crystal fields. No reliable simulation of these orbital effects can be envisaged without an independent determination of the *spin* and *orbital* components of the magnetization in magnetic materials. In the early nineties, X-ray Magnetic Circular Dichroism (XMCD) measurements made this possible within the limits of validity of the magneto-optical sum-rules [1–3]. This immediately boosted the development of sophisticated beamlines dedicated to *static* XMCD measurements in all synchrotron radiation facilities around the world, especially at the European Synchrotron Radiation Facility (ESRF) [4,5].

In 2005, X-ray Detected Magnetic Resonance (XDMR) emerged as a novel spectroscopy in which XMCD was used to *probe* the resonant precession of either spin or orbital magnetization components in a strong *pump* field typically oscillating at microwave frequencies. What stimulated our own interest in XDMR was the unique possibility to learn something new about *dynamical* aspects of orbital magnetism [6–10]. As a preamble, we like to show that much of the corresponding information is hidden in conventional electron magnetic resonance spectroscopies.

Let us start with the important remark made by Kittel [11] and others [12] that the torque equation **J̇** = **M***×* **H**, which correctly describes the precession of angular momenta of an isolated ion in the magnetic field **H**, does not hold true in a crystalline environment. If **J** denotes the total angular momentum per unit volume, then, magnetic dipole transitions are expected to induce a change Δ *J_z_* = Δ *J_z_^spin^* + Δ *J_z_^orbit^* + Δ *J_z_^lattice^* whereas the total magnetization *a priori* results from three contributions: **M** = **M***^spin^* +**M***^orbit^* +**M***^lattice^*. As suggested by Kittel, a major simplification arises if Δ *J_z_^orbit^* = −Δ *J_z_^lattice^*, e.g., due to a fast orbital relaxation process. Given that the lattice magnetization **M***^lattice^* is usually negligible, the torque equation then reduces to

(1) $$\frac{{dJ}^{spin}}{dt}=(M^{spin}+M^{orbit})\times H$$

We are left here with an oversimplified picture in which essentially the spin part of the angular momentum is precessing. Van Vleck [13] went to a similar picture using a microscopic theory that started from the quantum mechanical equation of motion: _1_*ħ* **Ṡ***_j_* = [ 
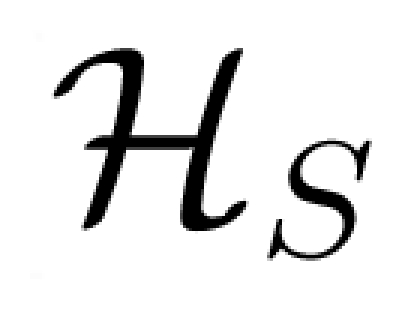
*,* **S***_j_* ] in which the spin Hamiltonian 
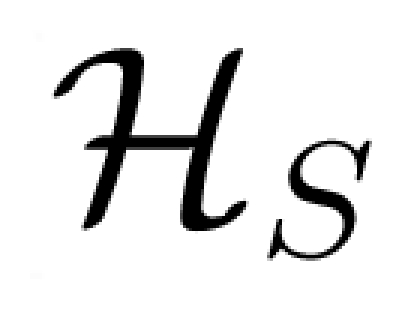
 was given the following form:

(2) $$\begin{array}{l} H_{S}=g\mu_{B}H\sum_{j}S_{j}^{z}+\sum_{k>j}J_{jk}S_{j}\cdot S_{k}+\sum_{k>j}D_{jk}[S_{j}\cdot S_{k}-3r_{jk}^{-2}(r_{jk}\cdot S_{j})(r_{jk}\cdot S_{k})]\\ +\sum_{k>j}E_{jk}{\left(r_{jk}\cdot S_{j}\right)}^{2}{\left(r_{jk}\cdot S_{k}\right)}^{2} \end{array}$$

in which *g* and *μ_B_* are standard notations for the spectroscopic splitting factor and the Bohr magneton, *J_jk_* being the isotropic exchange coupling constant between sites *j* and *k*. Whereas the first and second right-hand members of Equation 2 can readily be identified with the Zeeman and the isotropic Heisenberg exchange interactions respectively, the last two terms are analog to dipole-dipole and quadrupole interactions except that *D_jk_* and *E_jk_* can depart a lot from the expected values for dipolar/quadrupolar couplings between spins located at the *j, k* sites. This is precisely where spin-orbit interaction and orbital valence effects are taken into account to yield *pseudo*-dipolar (*S* = 1/2) or *pseudo*-quadrupolar (*S >* 1/2) couplings [14]. These terms which are the main ingredients of the anisotropic exchange coupling may substantially shift the resonance and alter the linewidth. Typically, the deviation of the *g*-factor from 2 is often taken as the signature of a pseudo-dipolar coupling since (*g–*2) ≃ *λ*/Δ*E* where *λ* denotes the spin-orbit coupling factor and Δ*E* is the energy separation between the ground orbital level and higher levels. The distortion of the electronic cloud along the spin direction should scale like (*g–*2)^2^ for a pseudo-dipolar (superexchange) interaction and (*g–*2)^4^ for the pseudo-quadrupolar coupling [15].

Spin Hamiltonians have been extensively used for the analysis of electron magnetic resonance spectra [16,17]. For orbitally degenerate systems, the concept of *fictitious* angular momentum operator equivalents turned out to be most helpful. The strategy is to retain only a subset of low-lying electronic states [12,16]:

- For crystal fields weak compared to spin-orbit coupling, *J* is a good quantum number and the crystal field simply splits the relevant manifold according to site symmetry. Ions with half-integral *J*’s always have a double Kramer’s degeneracy and fictitious spins take the following values: 
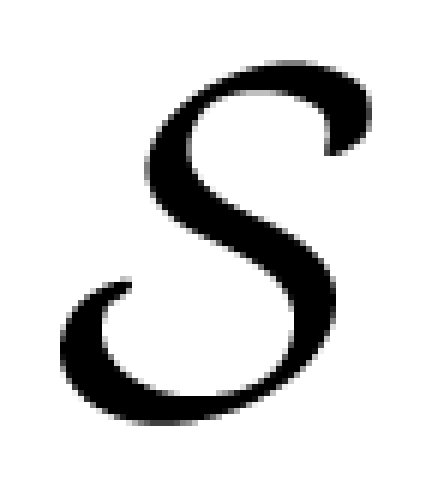
 = 0, 1/2, 1 or 3/2.
- If the crystal field is large compared to spin-orbit coupling, it splits the L-multiplet. If the lowest state is a singlet, the (quenched) orbital moment has no first order contribution. Recall that an orbitally threefold degenerate T-state mathematically is isomorphic to a P-state (*L* = 1): it can be described with a fictitious angular momentum 
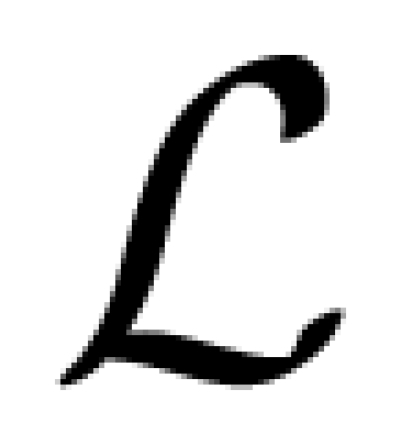
 which, in turn, couples to the spin angular momentum to yield a *fictitious*-spin 
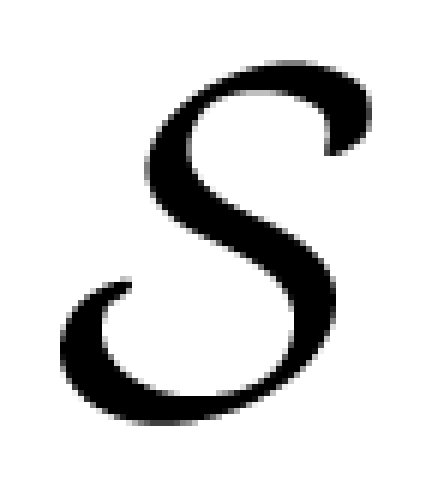
.

As noted by Blume *et al.* [12], a fictitious operator should be proportional to the expectation value of the true operator so-that one may write: ρ 
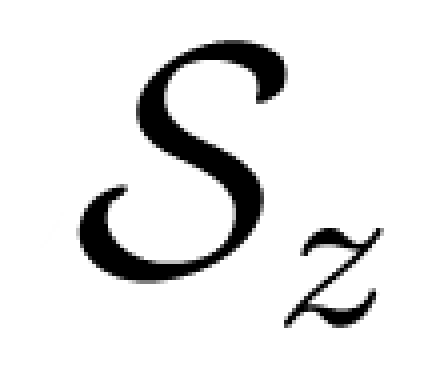
 = 〈*S_z_*〉 in which ρ ≠ 1 is a constant factor. Ultimately, a *fictitious* magnetization vector **M** can be defined which satisfies the equation of motion:

(3) $$1\hbar \frac{dM}{dt}=[M,H]≃[M,M\cdot H]$$

in which the Hamiltonian 
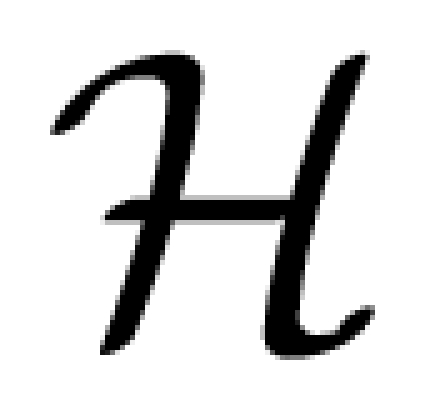
 reduces to a Zeeman term for EPR spectra whereas a compact formulation of the fictitious magnetization can be given using commonly accepted tensor notations: 
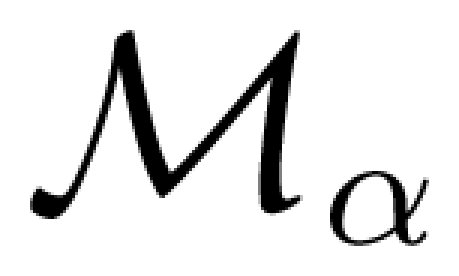
 = −*g_αα_μ_B_* 
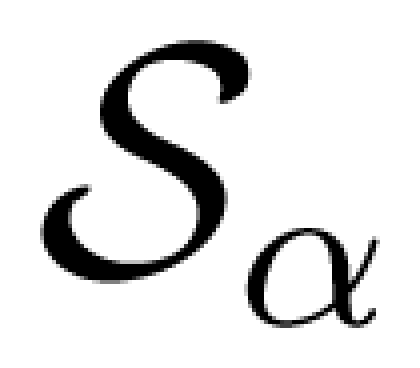
 with *α* = *x*, *y*, *z*. Concretely, the first or second order perturbations associated with orbital moments and spin-orbit interactions essentially affect the resonance through the spectroscopic splitting factor **g** which becomes a rank-2 tensor property. Recall that *det* [**g**] = *g_xx_g_yy_g_zz_* determines the helicity of the precession in the resonance field **H** [18]. Equation 3, however, does not tell us anything clear about the precession of *orbital* magnetization components, in particular in excited states.

Orbital effects add much complexity in those systems where the ground state is orbitally degenerated while magnetism is dominated by exchange interactions. Indeed the standard Heisenberg–Dirac form of exchange is not anymore appropriate and additional terms should be considered [19] if one wishes to describe the collective excitation of spin waves or study the magnetoelastic coupling effects which drive the spin-lattice relaxation processes [20]. One should also bear in mind that superexchange interaction has a dynamic character since it is produced by virtual hoping of electrons [22]: this is why the admixture of upper electronic states is often referred to as *orbital fluctuations* [21]. These fluctuations become most critical in Jahn–Teller ions in which orbital ordering effects were predicted independently by Kugel and Khomskii [23] and by Cyrot and Lyon-Caen [24]. In these recent years, there has been a renewed interest for the corresponding (Jahn–Teller) dynamical processes in connection with the excitation of orbital ordering waves (orbitons) [25]. The reality of such collective excitation processes seems to be well established for several antiferromagnetic oxides (e.g., in rare earth titanates, manganites and cuprates). Clearly, similar effects should take place in ferrimagnetic crystals as well.

It is the aim of the present paper to discuss the information content of XDMR spectra, emphasis being laid on the forced precession of orbital magnetization components. In Section 2, we briefly review the conceptual bases of XDMR and recast them in the framework of the linear and nonlinear theories of spin waves developed for FMR. What makes the difference between XDMR and FMR is clearly the element, edge and, in favorable cases, *site* selectivity of the XMCD probe. In Section 3, we focus on a series of XDMR spectra recorded on ferrimagnetic iron garnet films or single crystals in order to unravel various aspects of the precession dynamics of orbital magnetization components at the tetrahedral iron sites. Finally, in Section 4, we discuss how far it may be possible to extend XDMR experiments to paramagnetic complexes or antiferromagnetically (AFM) ordered materials.

## 2. Resonant Precession of Spin and Orbital Magnetization Components in Excited States

### 2.1. Specificity of the XMCD Probe

In XDMR, not only the frequency of the microwave *pump* field has to be adjusted in order to satisfy the usual conditions of magnetic resonance, but also the energy of the monochromatic, circularly polarized X-ray photons has to be carefully tuned in order to maximize the XMCD *probe* signal. This can happen only in the close vicinity of one of the multiple absorption edges which characterizes the photoionization of the core shells for a given absorbing element. In this respect, XDMR can be seen as an unusual *double resonance* experiment since it combines microwave Zeeman spectroscopy with an atomic, core level spectroscopy that is inherently element-selective. Recall that the X-ray absorption K-edge refers to the photoionization of a 1*s* deep core level, L_2_*_,_*_3_-edges being similarly associated with the photoionization of the spin-orbit split 2*p* core levels.

Edge-selectivity of XDMR measurements is a further advantage linked to the conservation of angular momentum in the photoionization process of deep atomic core levels, e.g., the 1*s* electrons of a K-shell or the 2*p* electrons of a L-shell. In the so-called *two-step* model [26], the angular momentum carried by a circularly polarized X-ray photon (+*ħ* for a right-handed circular polarization, −*ħ* for a left-handed circular polarization) is transferred to the excited photoelectron in a way that primarily depends on spin-orbit coupling in the excited core level. For L_2_*_,_*_3_ absorption edges that are split by spin-orbit, the Fano effect implies that part of the photon angular momentum is converted into spin *via* spin-orbit coupling (*ℓ* + *s* at the L_3_ edge; *ℓ – s* at the L_2_ edge). No such conversion is possible at a K-edge (or L_1_-edge) due to the absence of spin-orbit coupling in the core states and the photon angular momentum is entirely converted into *±ħ* orbital moments. The magnetic properties of the sample drive the second step: XMCD spectra simply reflect the difference in the density of final states that are allowed by the electric dipole (*E*1) or electric quadrupole (*E*2) selection rules owing to the symmetry of the initial core state. It immediately appears that an XMCD signal measured at a K-edge can only be assigned to the *orbital* polarization of the valence band. The interpretation of the XMCD spectra recorded at spin-orbit split edges is more complicated since one has to disentangle the respective contributions of spin and orbital polarizations. By summing up all integrated dichroisms of electrons originating from conjugated sub-levels, one would again measure the orbital polarization of the final states; in contrast the *difference* in the integrated dichroic intensities measured at the spin-orbit split L_3_ and L_2_ edges can only be caused by a spin imbalance in the empty states because the orbital momentum transferred to the photoelectron has implicitly the same sign at both edges. This is all the physical content of what is known as the magneto-optical sum rules for XMCD [1,2,26].

In XDMR, it is more appropriate to use a *differential* formulation of the XMCD sum rules as proposed by Strange [27] and others [28,29]. Ignoring first the contribution of electric quadrupole (*E*2) transitions, one may write for a K-edge [7,10] :

(4) $${\left[\Delta \sigma \right]}_{K}=3C_{p}{\left\{\frac{d}{dE}{\langle L_{z}\rangle }_{p}\right\}}_{\Delta E}=3C_{p}{\langle \ell_{z}\rangle }_{p}$$

in which [Δ*σ*]*_K_* is the difference in the absorption cross-sections of left- and right-circularly polarized X-rays in the vicinity of the K-edge, *C_p_* being a constant factor. Note that such a differential formulation refers to a *fixed* energy of the photoelectron: Δ*E* = *E_RX_* + *E*_0_ *– E_F_* in which *E_RX_*, *E*_0_ and *E_F_* respectively denote the energy of the X-ray photons, the binding energy and the reference Fermi level. Whereas 〈*L_z_* 〉*_p_* is the expectation value of the orbital angular momentum operator integrated over all excited states featuring *p*-type symmetry, 〈*ℓ_z_* 〉*_p_* defines the orbital polarization of *p*-projected densities of states (DOS) at energy Δ*E*. Taking into account the weaker electric quadrupole (*E*2) transitions simply results in mixing final states associated with atomic orbitals of different symmetry [7,10]:

(5) $$\langle \ell_{z}\rangle =\{{\langle \ell_{z}\rangle }_{4p}+ɛ{\langle \ell_{z}\rangle }_{3d}\}$$

One could easily extend this result to molecular complexes where the final states are described with molecular orbitals, e.g., when the absorbing atom is in a tetrahedral ligand field. In all cases, however, K-edge XDMR experiments will refer to the forced precession of some energy-dependent *orbital magnetization* component proportional to 〈*ℓ_z_*〉. XMCD cross-sections at L_2_*_,_*_3_-edges can similarly be related to differential operators involving spin and orbital magnetization DOS [7]:

(6) $$\begin{array}{l} {\left[\Delta \sigma \right]}_{L_{3}}=\frac{C_{d}}{3N_{b}}\frac{d}{d\Delta E}\{{\langle L_{z}\rangle }_{d}+\frac{2}{3}{\langle S_{z}\rangle }_{d}+\frac{7}{3}{\langle T_{z}\rangle }_{d}\}\\ =\frac{C_{d}}{3N_{b}}\{{\langle \ell_{z}\rangle }_{d}+\frac{2}{3}{\langle s_{z}\rangle }_{d}+\frac{7}{3}{\langle t_{z}\rangle }_{d}\} \end{array}$$

(7) $$\begin{array}{l} {\left[\Delta \sigma \right]}_{L_{2}}=\frac{C_{d}}{3N_{b}}\frac{d}{d\Delta E}\{{\langle L_{z}\rangle }_{d}-\frac{4}{3}{\langle S_{z}\rangle }_{d}-\frac{14}{3}{\langle T_{z}\rangle }_{d}\}\\ =\frac{C_{d}}{3N_{b}}\{{\langle \ell_{z}\rangle }_{d}-\frac{4}{3}{\langle s_{z}\rangle }_{d}-\frac{14}{3}{\langle t_{z}\rangle }_{d}\} \end{array}$$

in which *N_b_* *≃* 2 is the statistical branching ratio. Two distinct operators (*s_z_*, *t_z_*) are now needed to describe the spin dynamics. Recall that 〈*T_z_*〉 and 〈*t_z_*〉 reflect (to the lowest order) the asphericity of the spin magnetization due either to spin-orbit interactions or anisotropic charge distributions [26,30,31]. Though *T_z_* does not contribute to the Zeeman free energy, it contributes to the magnetocrystalline anisotropy free energy as pointed out by Van der Laan [31]. As long as the spin-orbit splitting remains small with respect to the crystal field splitting, **T** *≃* −2*/*7 **Q** · **u***_S_* in which **u***_S_* denotes the unit vector along the direction of the spin moment, whereas **Q** = **L**^2^ *–* 1*/*3*L*^2^ is the electric quadrupole operator which is a traceless spherical tensor of rank 2. In cubic crystals, **Q** vanishes so-that 〈*T_z_*〉 can reliably be neglected, especially for 3*d* transition elements. Anyhow, since the magnetocrystalline anisotropy free energy of ferrimagnetic iron garnets is fairly small, one can hardly expect a large contribution of 〈*T_z_*〉 and 〈*t_z_*〉 in these magnetic systems.

As opposed to electron magnetic resonance experiments which can be pumped with poorly energetic photons (*E ≤* 10 cm^−1^), XMCD measurements require highly energetic photons with wave numbers in excess of 10^7^ cm^−1^. Typically, the transition-time towards X-ray excited states is considerably shorter than the time-scale of spin-orbit interactions and of collective magnetic excitations involving exchange and dipole-dipole interactions. XMCD thus delivers a *snapshot* picture of the precession dynamics of spin and orbital magnetization components in *excited states* in the immediate surrounding of the X-ray absorbing atom.

### 2.2. Site-Selective Probe in Ferrimagnetic Iron Garnets

**Figure 1:**
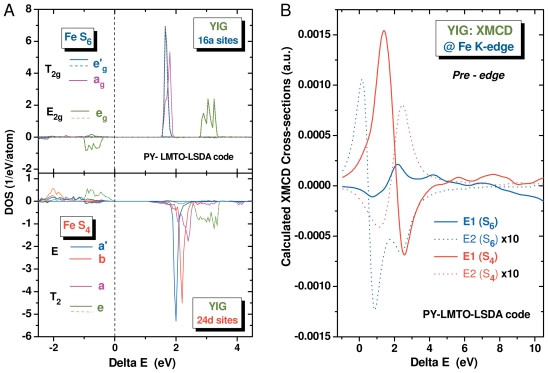
(**A**) *Ab initio* simulations of the spin polarized DOS projected on the irreducible representations of groups 
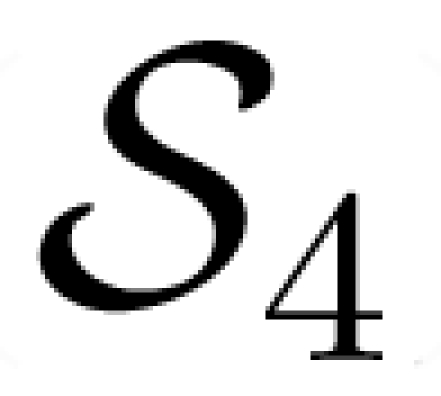
 and *S*_6_ at the Fe (24d) and Fe (16a) crystal sites in YIG: opposite signs were expected due to the antiferromagnetic coupling of the Fe cations in (24d) and (16a) sites; (**B**) Simulated XMCD spectra in the energy range of the Fe K-edge pre-peak. Note that the XMCD signal due to electric dipole transitions (*E*1) is much stronger at the tetrahedral ( 
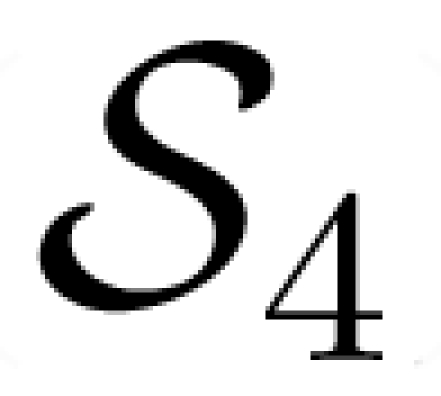
) than at the octahedral ( 
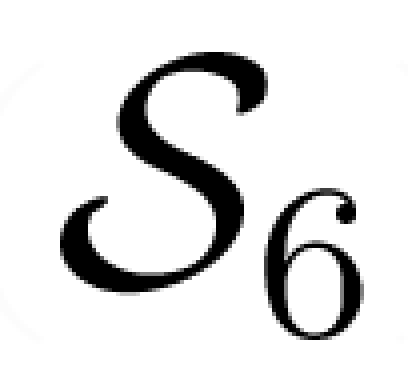
) coordination sites. The contributions of electric quadrupole transitions (*E*2) are one order of magnitude weaker.

This has some interesting implications illustrated with Figure 1A in which we have reproduced *ab initio* simulations of the spin-polarized DOS of yttrium iron garnet (YIG) in the ferrimagnetic state. The latter simulations carried out with the PY-LMTO-LSDA code [32] show that the spin-polarized DOS have the opposite sign at the (24d) and (16a) crystal sites in which the iron ions have slightly distorted tetrahedral ( 
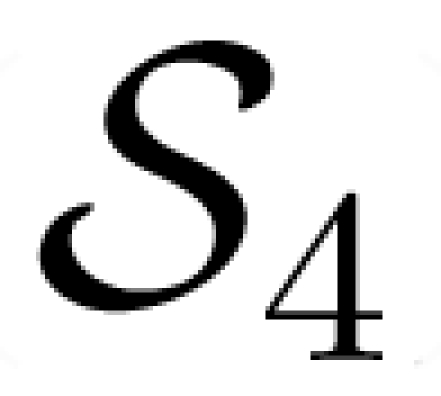
) and octahedral ( 
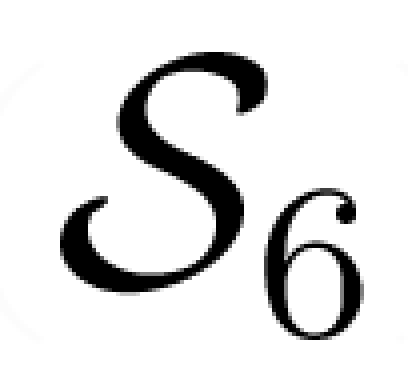
) coordination symmetry respectively. This is fully consistent with the antiferromagnetic coupling of the Fe ions in (24d) and (16a) sites [33]. Let us draw attention, however, onto the opposite sign of the spin polarized DOS in the filled states (Δ*E <* 0) and in the excited states (Δ*E >* 0) which belong to the same irreducible representation of groups 
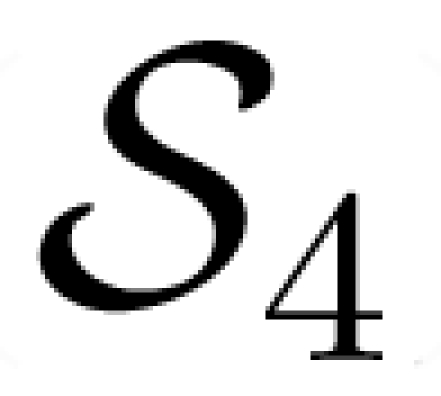
 or 
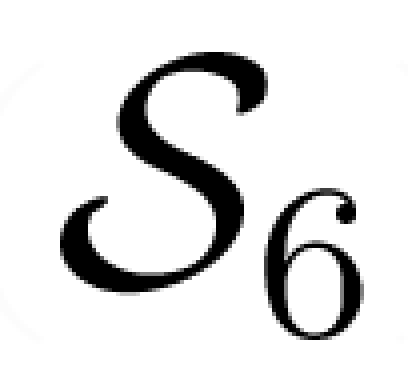
: this implies that the corresponding magnetization components should precess with the opposite helicity in the ground and excited states.

We have also reproduced in Figure 1B the XMCD spectra that can be deduced from the spin-polarized DOS of Figure 1A for both electric dipole (*E*1) and quadrupole (*E*2) transitions. The site-resolved XMCD spectra confirm the existence of a quite significant orbital magnetization component 〈*ℓ_z_*〉 at the tetrahedral iron sites: this is because *E*1 transitions are allowed from the 1*s* core level to final states which belong to the same representations (*b*, *e*) of group 
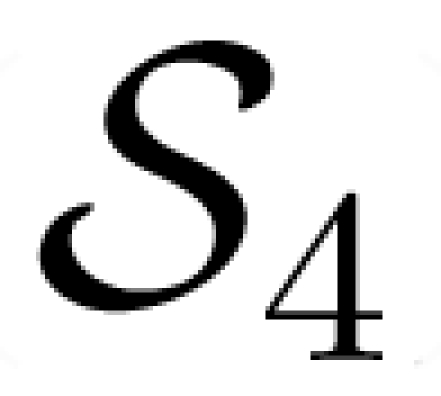
 as the 4*p* atomic orbitals. In contrast, transitions from the 1*s* core level to final states belonging to the *a_g_* or *e_g_* representations are allowed only by *E*2 transitions which have a much lower contribution to the XMCD cross-sections. In other terms, XDMR spectra recorded at the maximum intensity of the Fe K-edge XMCD spectrum will benefit from a strong site-selectivity favoring the 
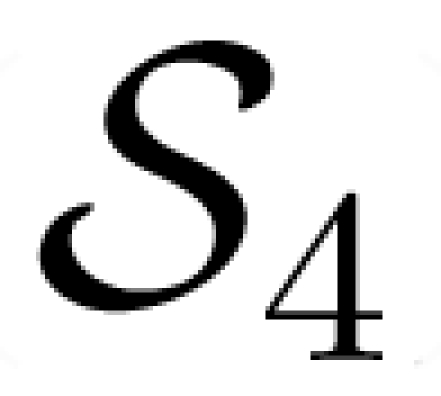
 Fe sites: this is a considerable advantage over FMR. Recall, however, that site-selectivity is lost if the XDMR spectra are collected at the L_2_*_,_*_3_ edges of iron since 2*p* → 3*d* transitions are dipole allowed at both sites.

### 2.3. Steady-State, Uniform Mode of Precession

Let us associate a fictitious, *local* magnetization component **M**^(^*^ℓ,s^*^)^ to 〈*ℓ_z_*〉 and 〈*s_z_*〉 respectively. The precession of **M**^(^*^ℓ,s^*^)^ can indeed be described either by Equation 3 or a classical torque equation. Regarding ferromagnetically ordered materials, the Landau–Lifshitz–Gilbert or the Bloch–Bloembergen torque equations are most appropriate even though they are purely phenomenological. The Landau–Lifshitz–Gilbert equation has a rather simple formulation for infinite thin films, *i.e.*, slabs with vanishing aspect ratio. Defining the local magnetization vectorMby its polar and azimuthal angles (θ*, φ*) and its norm *M*, one obtains the coupled equations [7]:

(8) $$(1+\alpha_{G}^{2})\frac{d\theta }{dt}=-\frac{\gamma }{M_{s}}\left(\alpha_{G}\frac{\partial F}{\partial \theta }+\frac{1}{\text{sin}\;\theta }\frac{\partial F}{\partial \varphi }\right)$$

(9) $$(1+\alpha_{G}^{2})\frac{d\varphi }{dt}=+\frac{\gamma }{M_{s}}\left(\frac{1}{\text{sin}\;\theta }\frac{\partial F}{\partial \theta }-\frac{\alpha_{G}}{{\text{sin}}^{2}\;\theta }\frac{\partial F}{\partial \varphi }\right)$$

in which the magnetic free energy *F* encompasses the Zeeman term (*F_Z_*), the magnetocrystalline anisotropy (*F_A_*) as well as the demagnetizing (*F_D_*) free energy responsible for the shape anisotropy. In equations 8 and 9, *α_G_* classically denotes the Gilbert damping parameter, *γ* = *gμ_B_/ħ*, whereas *M_s_* is the length of the fictitious magnetization component at equilibrium. Since the norm of the magnetization vector is not invariant in the Bloch–Bloembergen model, a third equation is needed [7]:

(10) $$\frac{\partial M}{\partial t}=-\omega_{r}[\frac{M_{s}}{H_{eq}}\frac{\partial F}{\partial M}-M]$$

in which *ω_r_* refers to a longitudinal relaxation frequency traditionally related to the spin-lattice relaxation time T_1_. It will become clear later on that the transverse relaxation time T_2_ is affected by additional relaxation processes involving a redistribution of the absorbed energy among all degrees of freedom of the whole spin system.

**Figure 2:**
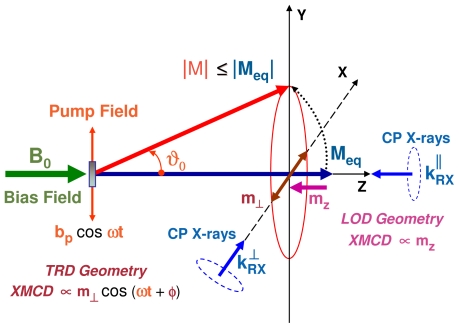
XDMR in transverse pumping mode with either longitudinal or transverse detection geometries for near perpendicular magnetization. The precession cone angle *θ*_0_ never exceeds a few degrees and was exaggerated for clarity. As compared to *m*_⊥_, *m_z_* is only a second order perturbation.

As illustrated with Figure 2, XDMR spectra can be recorded in two distinct geometries:

- In the *transverse* geometry (TRD), the wavevector **k***_RX_*^⊥^ of the incident, circularly polarized X-rays is set perpendicular to both the static bias field **B**_0_ and the oscillating pump field **b***_p_*: the XMCD probe is then proportional to the transverse magnetization *m*_⊥_ which oscillates at microwave frequency.
- In the *longitudinal* geometry (LOD), the X-ray wavevector **k***_RX_^||^* is set parallel to the static bias field **B**_0_. If one assumes that *M_s_* = *|***M***_eq_|*, then there is a *steady-state* change *m_z_* of the projection of the magnetization along the *Z* axis and there should be a time-invariant XMCD signal ∝ *m_z_*. Unfortunately, *m_z_* is only a second order effect with respect to the opening angle of precession (*θ*_0_) and *m*_⊥_. Moreover, any information regarding the phase or helicity of the precession gets lost.

For a ferromagnetic thin film with uniaxial anisotropy and perpendicular magnetization, the opening angle of precession *θ*_0_ is a constant of motion that characterizes the precession dynamics: it can be determined by combining together XDMR and *static* XMCD cross-sections [7,10]. For XDMR measurements in LOD geometry:

(11) $$[\Delta \sigma_{XDMR}(k_{RX}^{\left|\right|})]/[\Delta \sigma_{XMCD}(k_{RX}^{\left|\right|})]≃-1/2\;{\text{tan}}^{2}\;\theta_{0}$$

whereas in the TRD geometry:

(12) $$\left[\Delta \sigma_{XDMR}(k_{RX}^{⊥})]/[\Delta \sigma_{XMCD}(k_{RX}^{\left|\right|})]≃\text{tan}\;\theta_{0}\;\text{sin}[\omega t+\varphi_{0}\right]$$

The Landau–Lifshitz–Gilbert equations lead to the following resonance equations for *θ*_0_:

(13) $${\text{tan}}^{2}\;\theta_{0}=\frac{{\left(\gamma b_{cp}\right)}^{2}}{P_{0}^{2}+{\left(Q_{0}\;\text{cos}\;\theta_{0}\right)}^{2}}$$

in which *b_cp_* denotes the circular component of the microwave pump field and :

$$P_{0}=-\omega_{p}+\gamma B_{0}+\gamma [B_{u}-\frac{2}{3}B_{A1}(1-\frac{7}{4}\;{\text{sin}}^{2}\;\theta_{0})]\;\text{cos}\;\theta_{0}$$

where *B_u_* and *B_A_*_1_ refer to the uniaxial demagnetizing field and the cylindrical component of the magnetocrystalline anisotropy respectively. On the other hand, *Q*_0_ = −*α_G_ω_p_* in which the Gilbert’s damping factor *α_G_* is of the order of 6*×*10^−6^ for high quality YIG films. Similarly, one would show that:

$$\text{tan}\;\varphi_{0}=\frac{Q_{0}\;\text{cos}\;\theta_{0}}{P_{0}}$$

In the low microwave power limit, *i.e.*, when cos *θ*_0_ → 1, the resonance condition *P*_0_ = 0 will converge towards the usual Lorentzian lineshape whereas *φ*_0_ → *π/*2 if *P*_0_ → 0. However, for pumping fields in excess of 100 mG or higher, Equation 13 yields a biquadratic equation in cos *θ*_0_ with multivalued (unstable) solutions that characterizes the so-called *foldover* regime in which field-swept spectra exhibit a strongly hysteretic shape with a large foldover jump. This is very often the case in XDMR experiments carried out in LOD geometry because the pumping power has to be increased in order to get a chance to detect the weak XDMR signal which is only a second order effect.

Clearly, in TRD geometry, the oscillating signal Δ*σ_XDMR_*(**k***_RX_* *^⊥^*) varies linearly with the microwave pump field *b_p_*, whereas, in LOD geometry, the steady state signal Δ*σ_XDMR_*(**k***_RX_* *^||^*) is proportional to the incident microwave power *∝ b_p_* ^2^. At this stage, one should keep in mind that Δ*σ_XDMR_* as well as Δ*σ_XMCD_* are time-reversal *odd* observable properties which change their sign when the direction of *B*_0_ is inverted, independently of the oscillating pump field. Indeed, the normalized ratios (11)–(12) are time-reversal even: regarding the TRD geometry, this implies that, under time-reversal, the precession helicity (as well as *B*_0_) should be inverted in order to keep the product *ωt* invariant.

### 2.4. Precession Eigen Modes and Spin Waves

In magnetically ordered systems, the precession is driven by non-uniform, time-dependent fields **h**(**r***, t*) which do not simply reduce to the external pump field. This causes a number of orthogonal *eigen modes* to be excited in which all spins precess with the same frequency (*ω*) but not with the same phase. In this respect, dipole-dipole interactions -which are responsible for the demagnetizing field- open a whole band of magnons and make the degeneration of the uniform mode possible [38]. Let us emphasize that orbital magnetization components*M*^(^*^ℓ^*^)^ directly experience dipole-dipole interactions.

#### 2.4.1. Magnetostatic Walker’s Modes

In this regime, **h**(**r***, t*) follows Maxwell’s equations in the magnetostatic limit which means that the time-dependence of the electric field can be neglected [34]: *▿ ×* **h** = **0** ; *▿* · [*μ̄*]**h** = **0**. The first equation yields: **h** = −*▿ψ*(**r***, t*) and the second: *▿* · *μ̄ ▿ψ*(**r***, t*) = 0. These are Walker’s Equations. For a plane wave such as: *ψ ∝* exp[*i***k** · **r**], the Landau–Lifshitz–Gilbert and Walker’s Equations can be solved simultaneously only if a *dispersion* relation is satisfied [34]. For an infinite ferromagnetic medium:

(14) $$\omega_{\left|\right|}\leq \omega_{k}={\left[\omega_{H}\cdot (\omega_{H}+\omega_{M}\;{\text{sin}}^{2}\;\theta_{k})\right]}^{1/2}\leq \omega_{⊥}$$

in which *ω_H_* = *γ*(*B*_0_ *– N_z_M_s_* + *B_A_*_1_) and *ω_M_* = 4*πgμ_B_M_s_*; here, *N_z_* denotes the axial component of Kittel’s demagnetizing tensor. Typically, *ω_||_* and *ω_⊥_* are the resonance frequencies (*ω*) obtained for *θ_k_* = 0 or *θ_k_* = *π/*2 respectively, *i.e.*, when the wavevector **k** is either parallel or perpendicular to the direction of the bias field *B*_0_. Note that Equation 14 does not depend on the magnitude of *k* = *|***k***|* and thus, the group velocity *v_g_* = *∂ω/∂k* is zero. This degeneracy is removed if one takes into account either exchange interactions as discussed in the next section, or the sample finite boundaries. This is crucial for thin films in which the film thickness is usually much smaller than the microwave wavelength. Typically, for YIG thin films, the uniform mode exhibits a large number of sharp, discrete satellite resonances mostly assigned to *bulk* magnetostatic standing waves commonly referred to as *forward* or *backward* magnetostatic spin waves (MSW) depending whether *v_g_* is positive or negative [10]. The theory predicts as well the propagation of *surface* magnetostatic waves, the existence of which has found major applications in microwave technology.

We like to draw attention onto a further point. In addition to circularly polarized precession modes, elliptically polarized modes can be excited as well for which *v_g_* ≠ 0. In such a case, magnetostatic spin waves associated with wavevectors +**k** and −**k** have opposite helicity, the precessing moments being associated with **m_k_** and m^*^_−_**_k_** respectively. This is getting particularly important in magnetic materials featuring a large magnetic anisotropy [35]. Since the microwave pump field is most often linearly polarized, both Larmor and anti-Larmor precessions are co-excited, as confirmed by recent FMR experiments which made use of an ultra-sensitive mechanical detection [36].

### 2.4.2. Exchange Spin Waves

It is preferable to reformulate the Landau–Lifshitz–Gilbert equations as a system of canonical Hamiltonian equations for spin wave amplitudes (*c_k_*,c^*^_k_). Such a classical formulation strictly parallels the canonical transformations used by Holtstein and Primakoff in their quantum theory of spin-waves [37–39]. The equation of motion for each spin-wave amplitude *c_k_* has the form [41]:

(15) $$\frac{\partial c_{k}}{\partial t}=\omega_{r_{k}}c_{k}=-i\frac{\delta H(c_{k},c_{k}^{*})}{\delta c_{k}^{*}}$$

in which the relaxation frequency (*ω_r_k__* ) for mode *k* can be easily converted into the spin-wave full linewidth (Δ*H_k_*) using the relationship: *ω_r_k__* = *γ*Δ*H_k_/*2. Notice that the right-hand member of Equation 15 is expressed as a *functional* derivative of the Hamiltonian function 
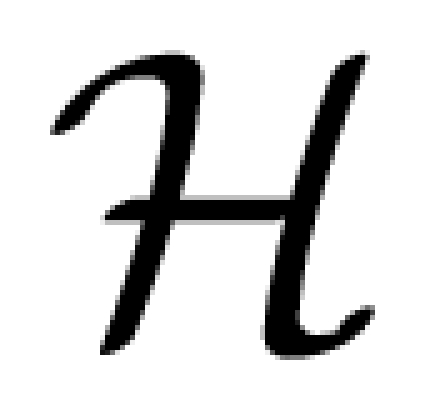
. Usually, spin-wave excitations in ferromagnets are sufficiently small that 
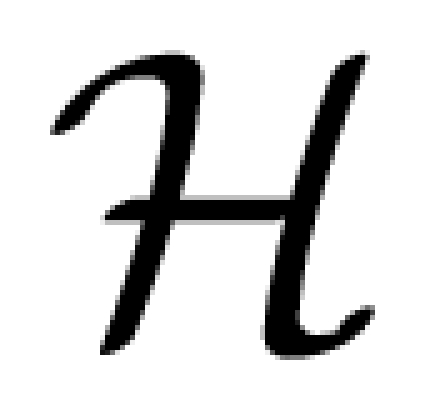
 can be expanded in a Taylor series in powers of *c_k_* and c_k_^*^:

(16) $$H(c_{k},c_{k}^{*})=\sum_{k}\omega_{k}c_{k}c_{k}^{*}+H_{p1}+H_{p2}+H_{int}$$

The first term in the right-hand side of Equation 16 is often denoted 
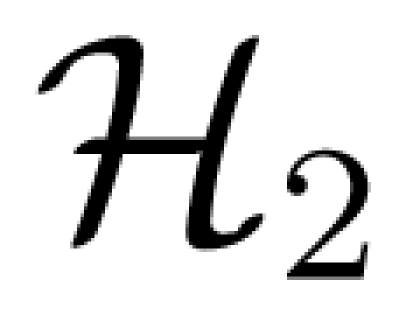
 but it corresponds to the *linear* theory of spin waves: it yields the energy of non-interacting modes with the dispersion law : *ω_k_* = [*A_k_*^2^ *– |B_k_|*^2^]^1^*^/^*^2^ in which:

(17) $$A_{k}=\omega_{H}+\eta_{ex}\omega_{M}k^{2}+|B_{k}|$$

(18) $$B_{k}=\frac{1}{2}\omega_{M}\;{\text{sin}}^{2}\;\theta_{k}exp(2i\varphi_{k})$$

where *η_ex_* is the exchange stiffness constant. Moreover, *θ_k_* and *φ_k_* refer to the polar and azimuthal angles of the wavevector **k** in spherical coordinates, taking the *z* axis parallel to the direction of the equilibrium magnetization **M***_eq_*. Recall that a precession mode becomes elliptical as soon as *|B_k_| ≠* 0 [20].

The next terms refer to the perturbation induced by the microwave pump field. The first one ( 
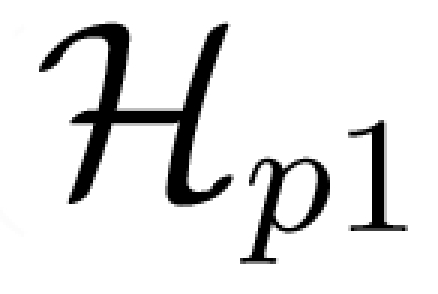
) arises from the Zeeman interaction with the *transverse* field component **b***_⊥_*:

(19) $$H_{p1}=\hbar (\gamma b_{⊥}){\left\{\frac{M_{s}V}{2g\mu_{B}}\right\}}^{1/2}(c_{0}e^{-i\omega_{p}t}+h.c.)$$

which causes the resonant excitation of the uniform mode (*k* = 0) in FMR. The nonlinear term 
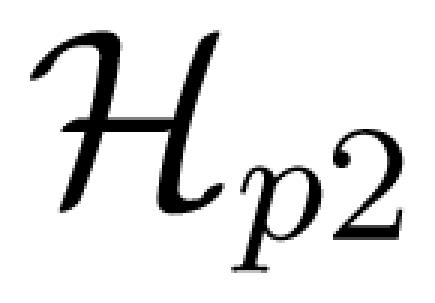
 is responsible for the spin wave instability in *parallel* pumping [43]:

(20) $$H_{p2}≃\hbar (\gamma b_{\left|\right|})e^{-i\omega_{p}t}\sum_{k}\frac{B_{k}}{4\omega_{k}}c_{k}c_{-k}+h.c.$$

It causes the parametric excitation of a pair of spin waves satisfying the conservation laws: *ω_p_* = *ω*_1_+*ω*_2_ and *k*_1_ = −*k*_2_ = *k*.

All information about the interaction of spin-waves between themselves is contained in the perturbation term 
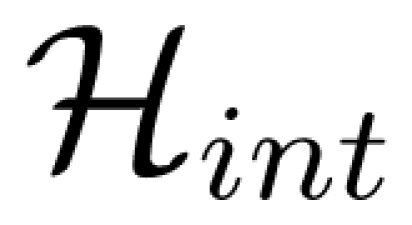
 = 
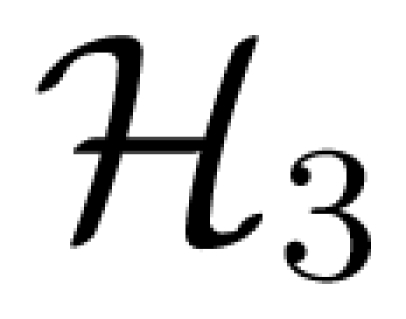
 + 
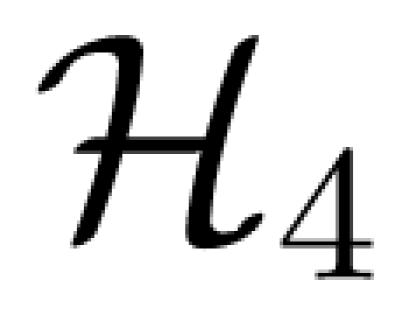
 with [39]:

(21) $$H_{3}=\sum_{k_{1},k_{2},k_{3}}\left[S_{k_{2},k_{3},k_{1}}c_{1}c_{2}^{*}c_{3}^{*}+S_{k_{2},k_{3},k_{1}}^{*}c_{1}^{*}c_{2}c_{3}]\times \delta (k_{1}-k_{2}-k_{3}\right)$$

(22) $$H_{4}=\sum_{k_{1},k_{2},k_{3},k_{4}}\left[S_{k_{3},k_{4},k_{1},k_{2}}c_{1}c_{2}c_{3}^{*}c_{4}^{*}+h.c.]\times \delta (k_{1}+k_{2}-k_{3}-k_{4}\right)$$

In the quasi-particle language of second quantification, 
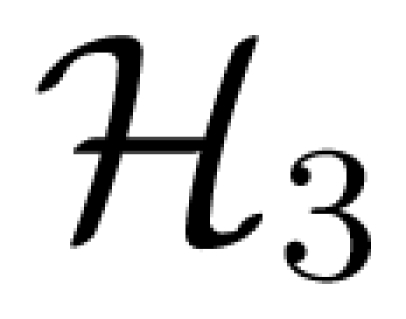
 and 
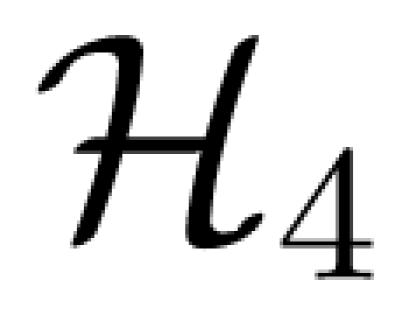
 describe three- and four-magnon interactions respectively. Three-magnon processes, in either the confluent or splitting modes, arise not only from long range dipole-dipole interactions as first recognized by Akhiezer [40], but also from short range pseudo-dipolar interactions [14]. Energy conservation implies that: *ω*_1_ = *ω*_2_ + *ω*_3_ for a 3-magnon splitting process, whereas (*ω*_1_ + *ω*_2_) = (*ω*_3_ + *ω*_4_) in a 4-magnon scattering process. Even though 
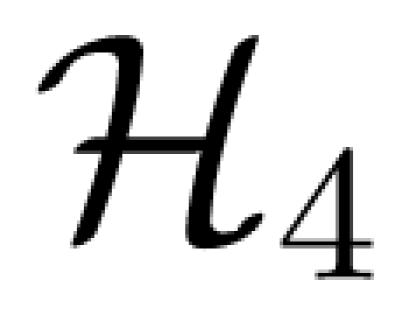
 refers to a higher order perturbation, the 4-magnon scattering process is the corner stone of all nonlinear theories of spin waves [41] because exchange may largely dominate over dipole-dipole interactions in this term [39]. Given that the exchange part is *α* (*k*_1_ · *k*_2_)^2^, it immediately appears, however, that the exchange cross section is zero if either *k*_1_ or *k*_2_ is zero: in other terms, exchange *cannot* relax the uniform mode (*k* = 0). As pointed out by Keffer [39], this is consistent with the well known result (see for instance [42]) that the Heisenberg exchange Hamiltonian commutes with all vector components of the *total* spin operator: 
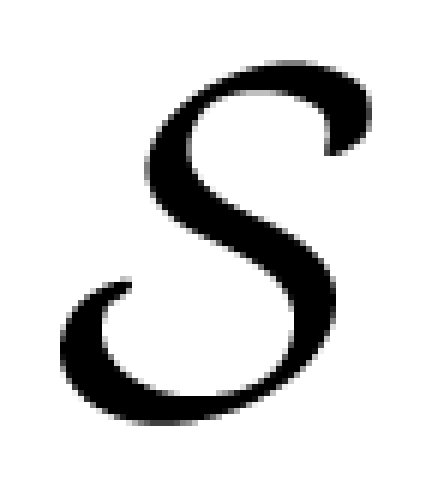
 = ∑*_i_* **S***_i_* so-that: [ 
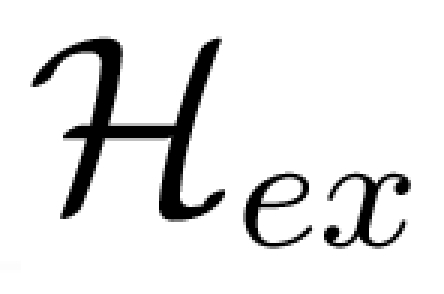
, 
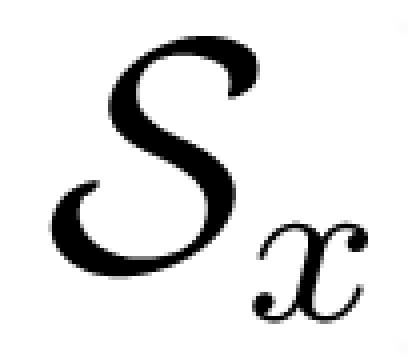
] = [ 
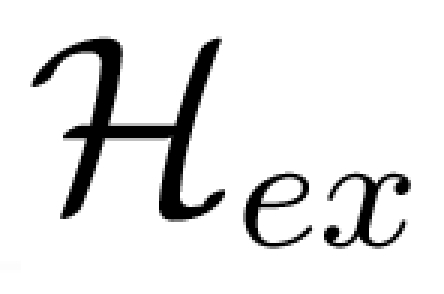
, 
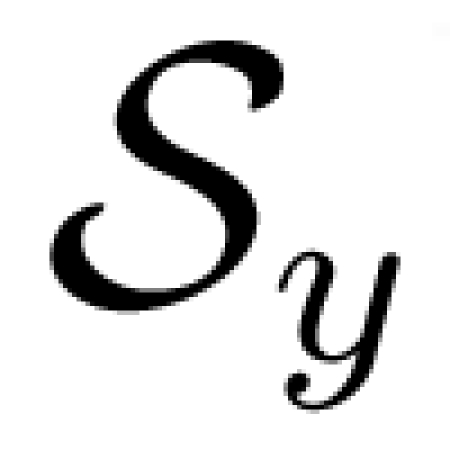
] = [ 
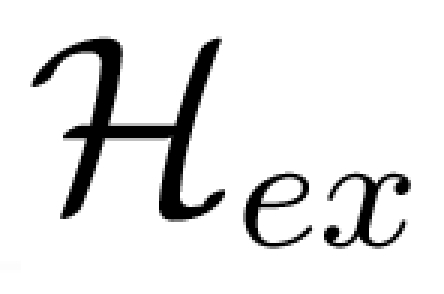
, 
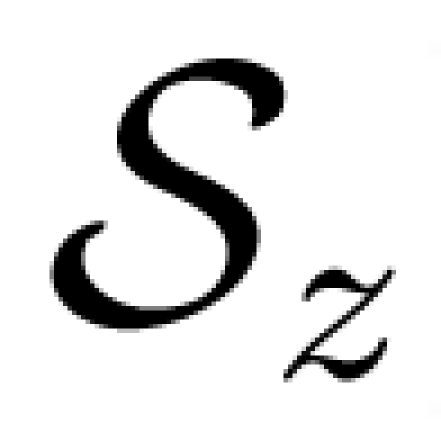
] = 0. In particular, this implies that the Heisenberg exchange Hamiltonian should also commute with the longitudinal (*z*) component of the magnetization: [ 
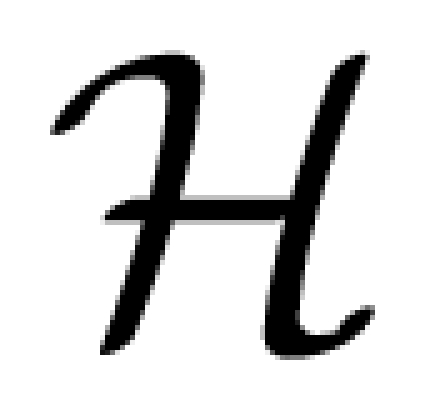
, 
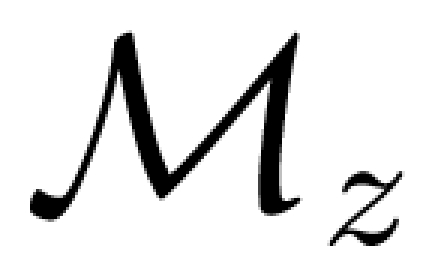
] = 0.

There is no doubt that the local spin magnetization components *M*^(^*^s^*^)^ probed by XDMR are directly affected by collective excitations of exchange spin waves. Even though exchange interactions should have no direct effect on orbital magnetization components *M*^(^*^ℓ^*^)^, one should not forget that *M*^(^*^ℓ^*^)^ and *M*^(^*^s^*^)^ are dynamically coupled by spin-orbit interactions at a time-scale much shorter than the time-scale of precession. Let us insist, however, that it cannot be taken yet as firmly established that *M*^(^*^ℓ^*^)^ couple to exchange spin waves.

#### 2.4.3. Mode Conversion in Relaxation Processes

Recognizing that dipolar interactions open a band of degenerate magnons is the clue to understand the relaxation mechanisms in FMR [38], since a uniform magnon (*k* = 0) can be annihilated with the simultaneous creation of one or more nonuniform magnons (*k ≠* 0): the latter *scattering* process was identified as the critical step controlling transverse relaxation processes, *i.e.*, T_2_ in the Bloch–Bloembergen model.

Statistical thermodynamics led to a pair of important equations which proved to be very helpful in discussing the relevance of various relaxation processes:

(23) $$M_{z}=M_{s}-g\mu_{B}V^{-1}\sum_{k}n_{k}$$

(24) $$|M|≅M_{s}-g\mu_{B}V^{-1}\sum_{k\neq 0}n_{k}$$

where *n_k_* is the number of magnons with wavevector *k* in volume *V*, including thermal magnons. Equation 23 implies that all types of magnons—including uniform ones—decrease *M_z_*; Equation 24 states that all spin waves but the uniform mode decrease *|M|*. Equations 23 and 24 are valid either for exchange or magnetostatic spin waves [20,38,39]. Clearly, the excitation of spin waves redistributes much of the microwave pumped energy within all internal degrees of freedom of the whole spin system before this energy is ultimately transferred to the lattice phonons.

### 2.5. Pseudo-Spin and Orbital Ordering Waves

Let us make a distinction between two different processes: (i) spin-orbit and dipole-dipole interactions can be strong enough to let the local orbital magnetization components *M*^(^*^ℓ^*^)^ couple to spin waves; (ii) energy can be redistributed within additional degrees of freedoms involving orbital ordering (O*_ord_*) in orbitally degenerate systems. In the first case we shall speak of *pseudo*-spin waves or *pseudo*-magnons. In the second, we have to deal with true orbital ordering waves or orbitons which can be best described using a formalism that was recently reviewed by Khaliullin [25].

The starting consideration is that both spin exchange and charge motion strongly depend on the orbital state in chemical bonding. One may easily admit that the interaction energy can hardly be optimized simultaneously for all bonds: this leads to peculiar frustrations, oscillations and quantum resonances among orbital bonds. Let us consider first the superexchange term of the Hamiltonian of a magnetic system which would be orbitally degenerate:

(25) $$H_{ex}=\sum_{i>j}\left[(S_{i}\cdot S_{j}+\frac{1}{4})J_{ij}^{\gamma }+\frac{1}{2}K_{ij}^{\gamma }\right]$$

For simplicity, we deliberately neglected Hund’s coupling in Equation 25. Orbital ordering effects are described by the operators *J_ij_^γ^* and*K_ij_^γ^*. Their formulation depends on the crystal structure and is different for doubly degenerate (*E* or *E_g_*) or triply degenerate states (*T*_2_ or *T*_2_*_g_*). The effects are most spectacular in the case of *E_g_*-states in a cubic crystal in which:

(26) $$J_{ij}^{\gamma }=J(\tau_{i}^{\gamma }\cdot \tau_{j}^{\gamma }-\frac{1}{2}\tau_{i}^{\gamma }-\frac{1}{2}\tau_{j}^{\gamma }+\frac{1}{4})$$

whereas: *K_ij_^γ^* = 0. In Equation 26,*J* = 4*t_h_*^2^*/U*, *t_h_* and *U* denoting respectively the hopping integral and the on-site Coulomb repulsion in the Hubbard model for two-fold degenerate states *E_g_*. The index *γ* of the operators *τ^γ^* specifies the orientation of the bond **r***_ij_* relative to the cubic axes *a*, *b* and *c*. Following Kugel and Khomskii [23]:

(27) $$\tau_{i}^{a,b}=\frac{1}{4}(-\sigma_{i}^{z}\pm \surd 3\sigma_{i}^{x})$$

(28) $$\tau_{i}^{c}=\frac{1}{2}\sigma_{i}^{z}$$

where *σ^x^* and *σ^z^* are Pauli matrices. According to usual conventions, *σ^z^* = 1 corresponds to orbital 3*d_x_*__2__*_–y_*__2__, whereas *σ^z^* = −1 refers to orbital 3*d*_3_*_z_*__2__*_–r_*__2__. Equations 25–28 therefore let us expect a strong interplay between spin and orbital degrees of freedom.

Regarding triply degenerate states (e.g., *T*_2_*_g_*) we shall again follow Kugel and Khomskii and replace the operators *τ_i_* with linear combinations of quadratic terms: [(*ℓ_z_*)*_i_*^2^ *–* 2*/*3], [(*ℓ_x_*)*_i_*^2^ *–* (*ℓ_y_*)*_i_*^2^] and [(*ℓ_x_ℓ_y_*)*_i_* + (*ℓ_y_ℓ_x_*)*_i_*], in which *ℓ_x_*, *ℓ_y_* and *ℓ_z_* are the components of a fictitious orbital angular momentum built up from the 3*d_xz_*, 3*d_yz_* and 3*d_xy_* transition metal orbitals. The superexchange Hamiltonian 
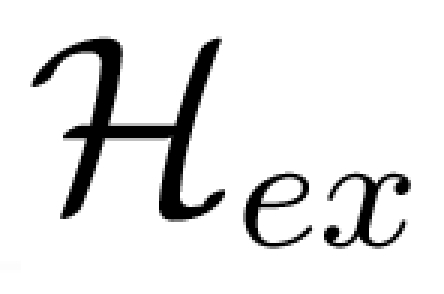
 is modulated by an operator 
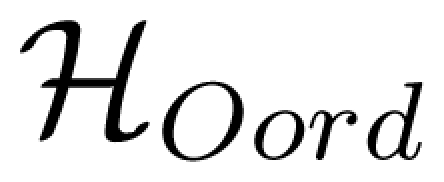
 which looks like the orbital quadrupole-quadrupole interaction ( 
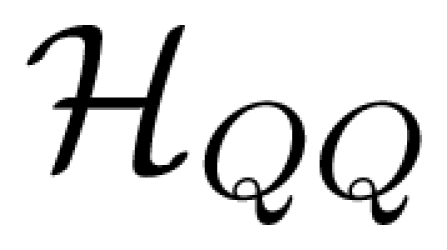
) between sites *i* and *j*, *i.e.*, the higher-order terms in the Van Vleck anisotropic exchange interaction. Note that 
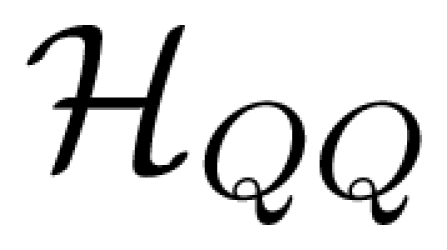
 also appears in the theory of the Jahn–Teller effect.

Collective excitations of the orbital ordering degrees of freedom were first envisaged by Cyrot and Lyon-Caen [24] who pointed out the marked similarity between the structure of 
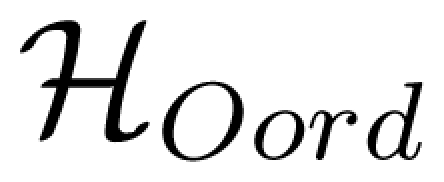
 and that of the spin wave Hamiltonian for antiferromagnetic interactions. In order to solve the superexchange problem of Equation 25, Khaliullin [25] proposed to split 
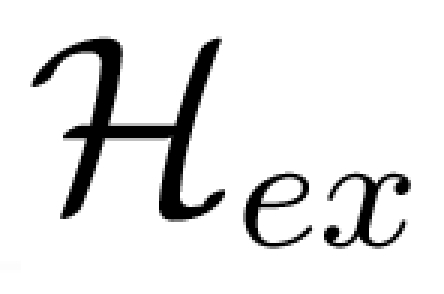
 in three terms:

(29) $$H_{spin}=\sum_{i>j}\langle J_{ij}^{\gamma }\rangle (S_{i}\cdot S_{j}+\frac{1}{4})$$

(30) $$H_{orb}=\sum_{i>j}\langle (S_{i}\cdot S_{j}+\frac{1}{4})\rangle \delta (J_{ij}^{\gamma })$$

(31) $$H_{int}=\sum_{i>j}\delta (S_{i}\cdot S_{j})\delta (J_{ij}^{\gamma })$$

in which *δJ* = *J –〈J〉*. Spin waves representations were systematically used for **S***_i_* in Equation 29 and, similarly, orbital waves were introduced for *τ_i_* in Equation 30. From a mathematical point of view, the easiest strategy to take into account the interaction Hamiltonian of Equation 31 is by exploiting diagram methods. Restricting ourselves here to three-bosons interactions, one may anticipate that an (excited) magnon could couple to an intermediate state containing one magnon plus one orbiton, whereas an orbital excitation (orbiton) could decay by a splitting into two magnons.

Regarding magnetic systems with orbitally degenerate ground states, Khaliullin went to the following conclusions: (i) fluctuations are enhanced in both spin and orbit subspaces; (ii) given the strong magnetic anisotropy of bonds, *quasi* 1-D orbital order is favored; (iii) joint spin-orbital fluctuations should significantly lower the ground state as first predicted by Cyrot and Lyon-Caen [24].

### 2.6. Spin Wave Instability Regime at High Pumping Power

#### 2.6.1. Transverse Pumping Geometry (*b_p_* *⊥ B*_0_)

Already in the late fifties, Suhl realized that a parametric amplification of spin-waves could cause a saturation of the precession cone angle (*θ*_0_) when the microwave pump field exceeded some critical threshold field *b_th_* [44,45]. He also pointed out that, under such conditions, the FMR lineshape would exhibit a foldover-like distortion [46]. Obviously, such effects should impact XDMR spectra as well.

**Figure 3:**
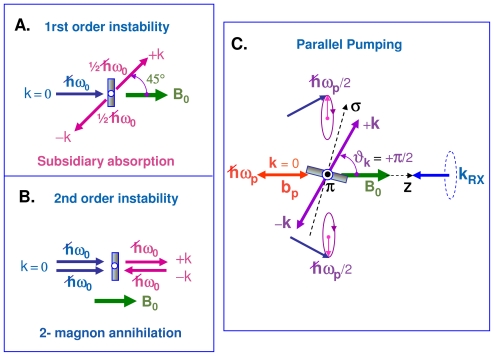
(**A**) First- and (**B**) second-order instability processes resulting in a conversion of uniform magnons into degenerated non-uniform magnons in transverse pumping geometry; (**C**) Parallel pumping geometry: direct excitation of a pair of non-uniform magnons with opposite wavevectors (*k*,*–k*) and opposite helicities. This non-linear, 2nd order process may contribute to a non-vanishing XDMR signal *only* in the longitudinal detection geometry (LOD).

There are two nonlinear processes by which the uniform precession mode can couple to degenerate spin waves. In the three-magnon process of Figure 3A, one uniform magnon splits into two magnons of lower energy but satisfying the conservation laws: *ω*_2_ = *ω*_3_ = *ω_p_/*2 and *k*_2_ = −*k*_3_ = *k*. In the four-wave scattering process of Figure 3B, two uniform magnons (*k*_3_ = *k*_4_ = 0) are destroyed whereas two degenerate magnons are created such that: *ω*_1_ = *ω*_2_ = *ω*_3_ = *ω*_4_ = *ω_p_* and *k*_2_ = −*k*_1_ = *k*. There is another difference between the first- and second-order processes: in the 3-magnon process, the pair of split magnons (+*k,* −*k*) propagates at *ca.* 45° from the bias field *B*_0_, whereas the pair of degenerate magnons created in a 4-magnon scattering process propagates along the direction of *B*_0_, *i.e.*, with *θ_k_* *≃* 0. The three-magnon process was shown to explain the *subsidiary* microwave absorption discovered by Damon and Bloembergen [48] at *ca. B*_0_ = *ω_p_/*2*γ*. Such an *off-resonance* process, however, cannot affect the XDMR spectra recorded on perpendicularly magnetized thin films because it is forbidden by energy conservation. The second order process that involves four magnons at the pumping frequency is always allowed and causes the main resonance to saturate at high microwave pumping power [20].

Instability arises when the rate of creation of a pair of degenerate spin-waves (+*k*, −*k*) exceeds their damping rate. For the 4-magnon process, growth and decay rates are given by [49]:

(32) $${\left[{\dot{n}}_{k}\right]}_{growth}=\frac{16\pi }{\hbar }|S_{0,0,k,-k}{|}^{2}n_{0}^{2}n_{k}\delta ({ɛ}_{f}-{ɛ}_{i})$$

(33) $${\left[{\dot{n}}_{k}\right]}_{decay}=-\frac{2\times n_{k}}{T_{2k}}=-n_{k}(\gamma \Delta H_{k})$$

in which *S*_0_*_,_*_0_*_,k,–k_* is the scattering factor introduced in Equation 22,*n*_0_ and *n_k_* being the number of uniform and nonuniform magnons with wavevector 0 and *k* respectively. Recall that the *δ*-function arises from the integral over the time-dependence of the initial (*i*) and final (*f*) spin states so that [39]:

(34) $$\delta ({ɛ}_{f}-{ɛ}_{i})=\frac{\gamma (\Delta H_{k})/\hbar }{\pi [{\left(2\omega -2\omega_{k}\right)}^{2}+{\left(\gamma \Delta H_{k}\right)}^{2}]}$$

Thus, one may predict the number of degenerate magnon pairs to grow exponentially when:

(35) $${\left[n_{0}\right]}^{2}\geq {\left\{\frac{\hbar^{2}[{\left(2\omega -2\omega_{k}\right)}^{2}+{\left(2\omega_{r_{k}}\right)}^{2}]}{16|S_{0,0,k,-k}{|}^{2}}\right\}}_{min}$$

whereas:

(36) $$n_{0}=\frac{MV}{2\hbar \gamma }\frac{{\left(\gamma b_{cp}\right)}^{2}}{{\left(\omega -\omega_{0}\right)}^{2}+\omega_{r_{0}}^{2}}$$

Here *ω_r_*__0__ and *ω_r_k__* denote the relaxation frequencies of the uniform and nonuniform modes respectively. Combining Equations 35 and 36 allows one to derive a threshold field (*b_cp_*)*_th–_*_2_ beyond which one should enter into the spin wave instability regime, provided that some useful expression of *S*_0_*_,_*_0_*_,k,–k_* is available. Restricting ourselves to the magnetic dipole-dipole (*dd*) part of 
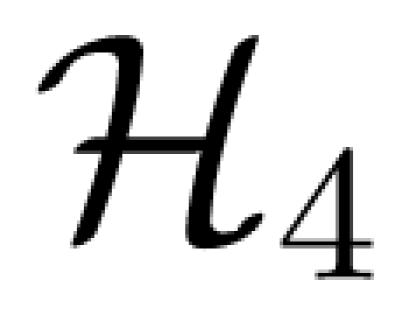
, one finally obtains for a perpendicularly magnetized thin film [20]:

(37) $${\left[b_{cp}\right]}_{th-2}≃\sqrt{\frac{\omega_{r_{k}}[{\left(\omega_{p}-\omega_{0}\right)}^{2}+\omega_{r_{0}}^{2}]}{\gamma^{2}\xi_{k}}}$$

in which *ζ_k_* is defined as:

$$\xi_{k}=\xi_{-k}=\frac{\omega_{k}+A_{k}}{4\omega_{k}}[\omega_{M}{\text{cos}}^{2}\theta_{k}+\eta_{ex}\omega_{M}k^{2}]$$

It immediately appears that *S*_0_*_,_*_0_*_,k,–k_^dd^* is maximum for *θ_k_* = 0: spin waves propagating along the normal to the film have therefore the lowest saturation threshold [20,39]. Clearly, one would like to raise the Suhl’s instability thresholds in order to increase the precession cone angle *θ*_0_. In practice, there are tricks to suppress the subsidiary absorption, but there is no way to remove the second-order instability [39].

#### 2.6.2. Parallel Pumping Geometry (*b_p_* *|| B*_0_)

As pointed out by Schlömann [43], spin-waves can be directly excited in the parallel pumping geometry illustrated with Figure 3C if there is a non-vanishing contribution of 
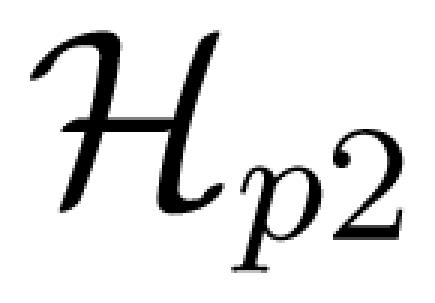
 defined in Equation 20. This requires spin waves with elliptic precession of the magnetization vector, as expected for tangentially magnetized films. The onset of this parametric process is obtained for a threshold field given by:

(38) $$b_{zth}≃\frac{\omega_{p}\Delta H_{k}}{\omega_{M}{\text{sin}}^{2}\theta_{k}}$$

in which Δ*H_k_* = 2*ω_r_k__/γ* is the full linewidth of mode *k*. Measuring *b_zth_* is an elegant way to determine the relaxation frequency (*ω_r_k__* ) of a selected mode [38]. Interestingly, nonuniform modes often exhibit much longer relaxation times than the uniform mode, *i.e.*, Δ*H_k_* *≪* Δ*H*_0_. As far as Δ*H_k_* does not depend on *k* or *θ_k_*, then *b_zth_* gets lower the closer *θ_k_* is to *π/*2.

Let us emphasize that the two magnons that are simultaneously excited have the same energy (1*/*2*ħω_p_*) but opposite *helicity*. Unfortunately, the XMCD signals due to the magnetization components *m_⊥_*(*k*) and *m_⊥_*(−*k*) cancel out since they are out of phase: this precludes the detection of the relevant XDMR spectra in a transverse detection geometry (TRD). Nevertheless, as illustrated with Figure 3C, a weak XDMR signal may well be detected in the longitudinal detection geometry (LOD) as it will be confirmed in Section 3.5: this is because *m_z_* (like *σ_XMCD_*) is a time-reversal odd observable but which remains unaffected by the helicity of the wavevectors *±***k**.

### 2.7. Instrumentation Constraints in XDMR Experiments

#### 2.7.1. Soft-XMCD *versus* Hard-XMCD Probes

One has to be aware that a specific beamline design and a fairly different instrumentation are required to carry out XMCD measurements with *“soft”* X-rays (*E <* 2 keV) as opposed to *“hard”* X-rays (*E ≥* 2 keV). Recall that soft-XMCD measurements are most often performed at the L-edges of 3*d* transition metals or at the M-edges of rare earths, and with some more difficulty, at the K-edge of oxygen. The wider energy range of hard X-rays (2–20 keV) makes it possible to record XMCD spectra at the K-edges of all transition metals, at the L-edges of rare-earths or 4*d* and 5*d* transition metals and at the M-edges of actinides. Unfortunately, the XMCD signal measured at K-edges of a 3*d* transitions metal is one or two orders of magnitude weaker than the XMCD signal measured at the corresponding L-edges with soft X-rays. This may well explain why many more attempts were made to record XDMR spectra in the soft X-ray range [50–60] than in the hard X-ray range [6–10].

Other considerations also come into play. In magnetic materials that suffer from large conductive losses (e.g., intermetallics or metallic multilayers with large magnetoresistance), the penetration of the microwave pump field is restricted to the skin depth which hardly exceeds 1 *μ*m. XDMR measurements will then suffer from a dramatic loss of sensitivity unless the penetration depth of the X-rays is made comparable with the skin depth. This is precisely the case with soft X-rays which are near-surface sensitive. Quite to the contrary, hard X-rays that are bulk sensitive, look more appropriate to probe insulators such as iron garnets and ferrites, or paramagnetic complexes.

#### 2.7.2. Combining Time and Frequency Domain Signals

As a preamble, let us mention that, regarding XMCD experiments carried out with hard X-rays, we do not measure directly the absorption cross-section but rather the total X-ray fluorescence yield caused by the photoionization of the deep core level. Let us stress, however, that there is no X-ray fluorescence detector that can measure a small dichroic signal oscillating at microwave frequencies as expected in the transverse detection geometry (TRD). At the ESRF, high quality XDMR spectra were recorded in the TRD geometry using a novel heterodyne detection [8,10]. The underlying concept can be easily catched by converting into the frequency domain the time-structure of the synchrotron radiation. Typically, the excited X-ray fluorescence intensity (*I_f_* (*t*)) consists of a series of discrete bunches, with a periodicity Δ*T* = 1*/RF* = 2.839 ns defined by the RF frequency (352.202 MHz) of the storage ring. Let us admit that all bunches have a gaussian shape with an average FWHM length of *ca.* 50 ps:

(39) $$I_{f}(t)=I_{f_{0}}\sum_{n}\delta (t-n\Delta T)⊗\frac{1}{\sigma \sqrt{2\pi }}\text{exp}-\frac{t^{2}}{2\sigma^{2}}$$

On Fourier-transforming *I_f_* (*t*), one obtains in the frequency domain a Gaussian envelope of harmonics of the RF frequency:

(40) $$H_{f}(F)=I_{f_{0}}RF\sum_{n}\delta (F-nRF)\text{exp}-2{\left(\pi \sigma F\right)}^{2}$$

One may easily check that the half-width at half maximum of the gaussian envelope: Δ*F*_1_*_/_*_2_ *≃* 25 *× RF* = 8.79 GHz falls in the microwave X-band. Since the ESRF storage ring directly provides us with a microwave *local oscillator* at a frequency close to the XDMR pumping frequency, we found it attractive to detect their low-frequency beating signal. A further gain in sensitivity was obtained by using a super-heterodyne detection scheme which exploits a 180° bi-phase modulation technique (bpsk: “bi-phase shift keying”) [10]. Defining the XDMR pumping frequency as *F_p_* = *N × RF* + *IF*, the superheterodyne detection consists in catching the modulation satellites at frequencies *IF ± F_bpsk_*. Nearly all XDMR spectra recorded so far at the ESRF were measured in the X-band using as reference frequency *F_N_* = 24 *× RF* = 8452.856 MHz, but our equipment can still be operated with *N ≤* 54 (*i.e.*, *F_N_* = 19018.927 MHz).

The ESRF heterodyne (or superheterodyne) detection scheme falls in the group of time-average measurement methods. Arena, Bailey *et al.* [50–55] preferred a time-resolved approach in which the pumping frequency is directly a low-order harmonics of the RF signal at the Advanced Photon Source at Argonne National Laboratory (USA). The XMCD signal is then sampled stroboscopically by the X-ray pulses. This method suits remarkably well to soft-XDMR experiments on metallic multilayers for which the pumping frequency (*F_p_* *<* 4 GHz) is restricted by skin depth considerations.

## 3. XDMR in Ferrimagnetic Iron Garnets

### 3.1. Selected Samples

With excellent insulator properties and no detectable magnetic disorder, yttrium iron garnet (YIG) has played a historical role in the promotion and understanding of FMR. It has a cubic structure (space group:Ia3̄d; group N°230), each unit cell consisting of eight formula units Y_3_[Fe_2_](Fe_3_)O_12_ [61]. This formulation emphasizes the role of the tetrahedral ( 
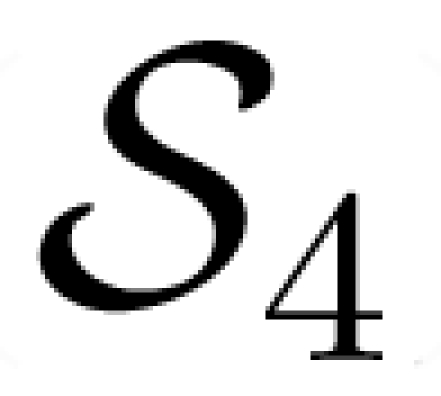
) and octahedral ( 
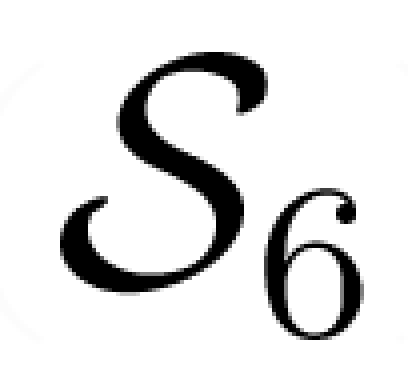
) iron sites. Below the magnetic ordering temperature (*T_c_* *≃* 550 K), the two Fe sublattices get magnetized antiparallel one to each other, with an unbalanced magnetization (*ca.* 5 *μ_B_*) in favor of the tetrahedral sites. The ferrimagnetic order is caused by a strong superexchange interaction between the two iron sublattices, the rather large Fe(16*a*)*–*O*–*Fe(24*d*) angles (126.6°) giving a clear indication that the wavefunctions of oxygen and iron have a substantial overlap. YIG is the generic term for a rich family of iron garnets in which yttrium can be substituted with rare earths in variable proportions.

In Section 3, we shall reproduce XDMR spectra collected essentially on two iron garnet films that were grown by liquid phase epitaxy on oriented gadolinium gallium garnet (GGG) substrates: **1** = Y_3_Fe_5_O_12_ (YIG # 520); **2** = [Y_1.3_La_0.47_Lu_1.3_]Fe_4.84_O_12_ (Y-La-LuIG). Note that in film **2**, “*diamagnetic*” (^1^*S*_0_) rare earth cations (La^3+^, Lu^3+^) substitute for Y^3+^. In Section 3.4, we shall also briefly discuss XDMR spectra recorded with a thin, polished platelet of gadolinium iron garnet (GdIG) single crystal.

### 3.2. XDMR Spectra of a YIG Thin Film Recorded in Transverse Detection Geometry

**Figure 4:**
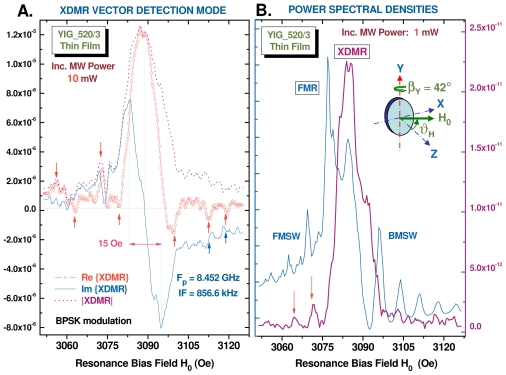
(**A**) Complex vector XDMR spectrum of film **1** recorded in transverse detection geometry (TRD) with a pumping power of 10mW. Arrows point to weak satellite resonances assigned to magnetostatic modes; (**B**) Comparison of FMR and XDMR PSD spectra that were simultaneously recorded again in TRD geometry but with a pumping power of only 1 mW. Note the fairly different lineshapes of the main resonances assigned to the uniform mode. Moreover, there is only a very weak contribution of the magnetostatic modes (BMSW) satellites in XDMR.

We like first to show that XDMR spectra of unprecedented quality can now be recorded in the transverse detection geometry (TRD) on YIG and related thin films, thanks to our superheterodyne detection scheme that relies on the powerful concepts of either bpsk or qpsk (bi- or quadrature phase shift keying) used in satellite telecommunications. A vector detection scheme also allows us to recover the phase information that is preserved in a TRD geometry. This is illustrated with Figure 4A in which the absorptive (*χ*″) and dispersive (*χ*′) XDMR components of film **1** can be identified with the real and imaginary parts of the vector detection scheme. A small (instrumentation dependent) phase-shift (ΔΦ *≃* 2°) was added in order to let *χ*′pass through zero when *χ*″is maximum as well as *|XDMR|*. In this experiment, the energy of the circularly polarized X-ray photons was tuned to the maximum of the Fe K-edge XMCD spectrum (*E*_1_ = 7113.91 eV) and the film was rotated by *β_Y_* *≃* 42^0^ in order to minimize the demagnetizing field anisotropy. We checked that the XDMR peak intensity varied linearly with the square root of the pumping power up to *ca.* 10 mW.

Arrows in Figure 4A point to weak satellite resonances assigned to either backward (BMSW) or forward (FMSW) magnetostatic spin waves [10]. This is a clear indication that, *locally*, the *orbital* magnetization components *M*^(^*^ℓ^*^)^ couple to non uniform magnetostatic spin waves through dipole-dipole interactions. Note that forward/backward MSW seem to have different phases. At this stage, one should realize that there is no chance to excite and detect standing waves resonances associated with magnetostatic modes unless there is a *net* transverse magnetization component interacting with both the microwave pump field and the circularly polarized X-rays: in the YIG thin film **1**, this can be envisaged only for standing waves of rather low order and featuring an *odd* number of semiperiods [62]. In this respect, we already discussed elsewhere [10] the reasons which let us expect the relative amplitude of the forward/backward MSW satellites to be much weaker in XDMR than in conventional FMR spectra.

In Figure 4B, we compare the FMR and XDMR power spectral density (PSD) spectra that were recorded simultaneously under a pumping power of only 1 mW. It immediately appears that the two PSD spectra do not peak at the same resonance field whereas the intensity of the sharp BMSW modes are considerably more intense in the FMR spectrum. Also the XDMR linewidth is definitely broader. Unfortunately, by the time of these early experiments, the limited number of channels in our vector spectrum analyzer prevented us from simultaneously recording both the FMR and XDMR spectra in a fully coherent *vector* mode. This technical problem was recently solved but no beamtime was allocated as yet to further tests.

### 3.3. Element Resolved XDMR of the Y-La-LuIG Film in Longitudinal Detection Geometry

Long before we started the XDMR experiments reported below, a *static* XMCD study of film **2** had revealed the presence of large *induced* spin components *〈s_z_〉* at the location of the “diamagnetic” trivalent cations La^3+^, Lu^3+^, Y^3+^ [33]. Technical arguments (e.g., circular polarization rates, X-ray fluorescence yields . . . ) convinced us that XDMR experiments carried out at the La L_2_*_,_*_3_-edges would be less beamtime-consuming than similar experiments at the low-energy Y L_2_*_,_*_3_-edges. Preliminary test experiments carried out on film **2** in longitudinal detection geometry (LOD) led to the rather unexpected result that, under perpendicular magnetization, the *apparent* precession cone angle of the orbital magnetization component *M*^(^*^ℓ^*^)^ measured at the Fe K-edge, *i.e.*, *θ*_0_^(^*^ℓ^*^)^ [*Fe*] = 13 *–* 19°, was much larger than the *apparent* precession cone angle of the spin magnetization component *M*^(^*^s^*^)^ measured at the La L_2_*_,_*_3_-edges, *i.e.*, *θ*_0_^(^*^s^*^)^ [*La*] = 4.7°. Even more puzzling was the large precession cone angle *θ*_0_^(^*^ℓ^*^)^ [*Fe*] measured for film **2**, especially if we compare it to the opening angle (*θ*_0_^(^*^ℓ^*^)^ [*Fe*] = 7.2°) measured for the YIG film **1** under identical experimental conditions. In contrast, *θ*_0_^(^*^s^*^)^ [*La*] was found to be slightly smaller than the cone angle measured at the Y L-edges in film **1** (*i.e.*, *θ*_0_^(^*^s^*^)^ [*Y*] = 5.9°).

**Figure 5:**
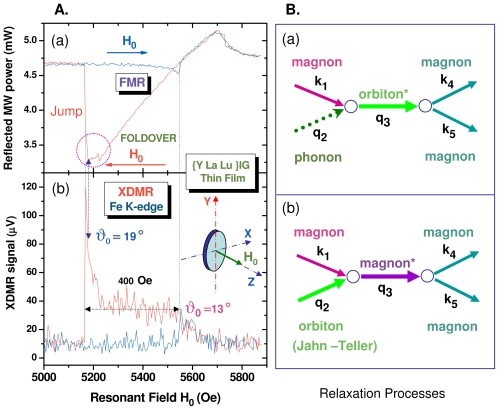
(**A**) Foldover distorted XDMR and FMR spectra of film **2** showing hysteretic profiles when the bias field is scanned up and down; the XDMR spectrum was recorded in longitudinal detection geometry (LOD) under a pumping power of 630 mW. Note the very sharp increase of the XDMR signal just before the foldover jump whereas the FMR spectrum seems to saturate; (**B**) Modified Kasuya–LeCraw relaxation mechanisms involving an orbiton either in a 3-boson de-excitation (**a**) or excitation (**b**) process.

We compare in Figures 5A the XDMR and FMR PSD spectra recorded simultaneously with the Y-La-LuIG film **2** under high pumping power (630 mW). The XDMR spectrum was again recorded in the longitudinal detection geometry (*k_RX_^||^* *|| B*_0_). The FMR and XDMR spectra both exhibit strong foldover distortions resulting into broad lineshapes (Δ*H*_0_ *≥* 400 Oe). Most intriguing to us was the very sharp increase of the XDMR signal just *before* the foldover jump in the downfield scan, in a range where the FMR absorption spectrum seems to saturate. Even far from the foldover jump, the precession cone angle (*θ*_0_^(^*^ℓ^*^)^ [*Fe*] *≃* 13°) is much too large to be realistic: this definitively ruled out the crude assumption that the length of the precessing moment (*|M*^(^*^ℓ^*^)^*|*) was invariant and convinced us to envisage the parametric excitation of nonuniform modes.

From Equation 24, one would expect the annihilation of two uniform magnons and the creation of two degenerate magnons to shorten the length of the magnetization vector *|***M**^(^*^ℓ^*^)^*|* and to increase the transverse relaxation rate 1*/T*_2_. Regarding Equation 23, one would guess that the four-magnon scattering process of Figure 3B should leave *M_z_*^(^*^ℓ^*^)^ unaffected since two uniform magnons are replaced by two degenerate magnons, so that the total number of magnons is left unchanged. This, however, does not hold true if the life-times of uniform and degenerate magnons are different. Schlömann [64] was the first to point out that, in the four-wave interaction process of Figure 3B, the damping rate of the spin waves (+*k*, −*k*) was artificially decreased. Typically, he introduced an *effective* damping rate:

(41) $${\tilde{\omega }}_{r_{k}}={\left(\omega_{r_{k}}^{2}-|S_{0,0,k,-k}{|}^{2}|b_{0}{|}^{4}\right)}^{1/2}$$

in which the amplitude of the uniform mode *|b*_0_*|*^2^ directly depends on the incident microwave power. Clearly, the higher the microwave power, the longer should be the lifetime of the pair of degenerate magnons. Equation 23 then predicts a rapid decrease of *M_z_*^(^*^ℓ^*^)^, *i.e.*, a sharp raise of *m_z_*^(^*^ℓ^*^)^ exactly as observed in Figure 5A. We still need to understand why this effect is so spectacular with film **2** but does not show up with film **1**, and why it does not affect in the same way the magnetization components probed at the iron or lanthanum sites. It is our interpretation that this has to do with a relaxation mechanism that selectively affect the orbital magnetization component *〈ℓ_z_〉* at the iron 
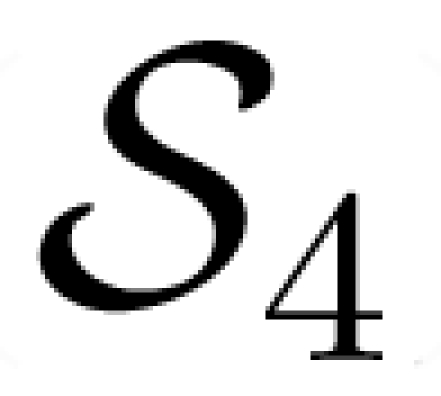
 site in the Y-La-LuIG film. Owing to the fact that the orbital magnetization components *〈ℓ_z_〉* are heavily quenched at the rare earth sites [33], no significant relaxation anomaly can be detected at the La or Lu L-edges. This nicely illustrates the different nature of the relaxation mechanisms at different magnetic sites.

In the Kasuya–LeCraw process which is regarded as the dominant spin-lattice relaxation process in YIG [20,63], it is postulated that the confluent scattering of a small **k** degenerate magnon with a phonon **q** can produce an excited magnon of much higher energy: the latter may decay through a cascade of three-magnon splitting processes now allowed by energy conservation. Since the net result is an increase of the total number of magnons, Equation 23 let us expect *M_z_* to decrease or *m_z_* to increase: of course, it would be totally erroneous to assign this effect to an (improbable) increase of the precession cone angle of the uniform mode in Figure 2. We have sketched in Figure 5B. two modified Kasuya–LeCraw mechanisms in which a cascade of two consecutive 3-boson processes may involve one orbiton either in a de-excitation process (a) or in the excitation process (b).

Mechanism (5B.b) deserves more attention in a context where valence or orbital fluctuations may cause a dynamical replacement of Fe^3+^ cations in either tetrahedral or octahedral sites with short living Jahn–Teller cations (Fe^4+^, Fe^2+^) that had unquenched orbital moments. Such valence fluctuations could be initiated or enhanced by the presence of small amounts of impurities (e.g., Pb^2+^) introduced during the growth of the film by liquid phase epitaxy. In other terms, the ^6^*S*_5_*_/_*_2_ configuration for iron may not be time-invariant. Recall that the effects induced by Fe^4+^, Fe^2+^ or other Jahn–Teller ions in doped YIG films have fed long debates that are not closed. In the case of film **2**, strong perturbations stem from the large size of the La^3+^ cations and of the small size of the Lu^3+^ cations which allow lutetium to partly replace iron in the octahedral sites. The non-uniform distribution of the rare earth cations induces fluctuations in the lattice parameter, *i.e.*, dynamical strains and stresses resulting in the excitation of magneto-elastic waves. This is supported by the much larger growth anisotropy of film **2** as compared to film **1**. Nevertheless, it would be desirable to lay deeper foundations to the mechanisms of Figure 5B. In particular, we are interested in establishing the link between this mechanism and the *slow* longitudinal relaxation theory pioneered in the early sixties by Van Vleck and others [66,67].

### 3.4. Anomalous Saturation of the Fe K-Edge XDMR Spectra of GdIG

The replacement of all Y^3+^ ions with Gd^3+^ (^8^*S*_7_*_/_*_2_) in the dodecahedral (24c) sites results in a more severe perturbation than that caused by the *pseudo*-diamagnetic cations La^3+^ or Lu^3+^. Even though YIG and GdIG have identical crystal structures and nearly the same Curie temperatures (*T_C_* = 551–556 K), their magnetic properties are fairly different due to the contribution of the *weakly* coupled Gd sublattice, the Gd spins getting fully ordered only below a low ordering temperature (*T_B_* *≃* 67 K) [68]. Above *T_B_*, the Gd magnetization can be described as a temperature-dependent Brillouin function for spin 7/2 in a field proportional to the net magnetization of the strongly coupled ferric ions. The most spectacular consequence is the existence of a compensation point, *i.e.*, a temperature (*T_cp_* = 286 K) at which the spontaneous magnetization of GdIG passes through zero [61]. Considerable changes in the FMR spectra take place at the compensation point. One of them is the inversion of the Larmor precession helicity on passing through the compensation point [69]. This prompted us to check whether this could be seen in the Fe K-edge XDMR spectra of GdIG.

**Figure 6:**
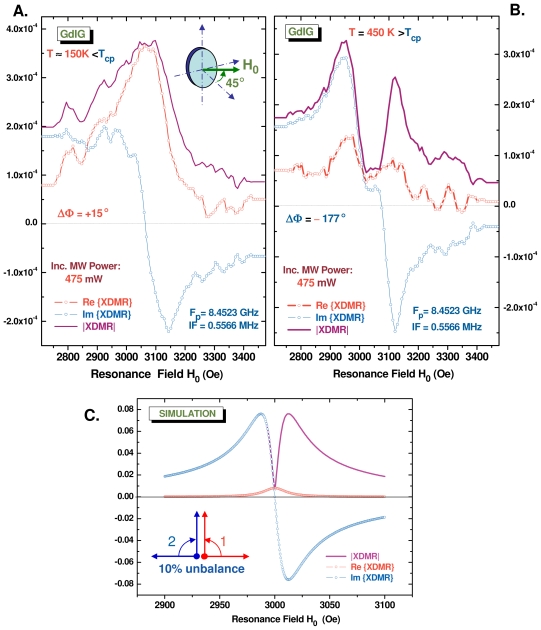
Complex vector XDMR spectra recorded in transverse detection geometry (TRD) with a thin single crystal platelet of GdIG cut parallel to the (110) plane: (**A**) *Below* the compensation point, *i.e.*, at *T ≃* 150 K *< T_cp_* = 286 K; (**B**) *Above* the compensation point, at *T* = 450 K *> T_cp_*. Note the very weak intensity of the absorptive part (*Re{*XDMR*}*) and the heavily distorted lineshape of the modulus spectrum (*|*XDMR*|*); (**C**) Complex vector XDMR spectra typically expected for two magnetization components precessing in phase but with opposite helicity. Note that the absorptive part (*Re{*XDMR*}*) again tends to vanish whereas the modulus spectrum (*|*XDMR*|*) exhibits a characteristic “dip” at resonance as also observed in (**B**).

We have reproduced in Figure 6 complex vector XDMR spectra of a thin disk of GdIG cut parallel to the (110) planes. These Fe K-edge XDMR spectra were recorded in transverse detection geometry (TRD). Since the normal to the disk was rotated by 45*°* with respect to the direction of *B*_0_, the contribution of the uniaxial anisotropy was heavily reduced. In Figure 6A, we display the XDMR spectra measured at *T ≃* 150 K, *i.e.*, at an intermediate temperature well above the ordering temperature *T_B_* but below the compensation temperature *T_cp_*; in Figure 6B, the XDMR spectra of the same sample were measured at *T* = 450 K, *i.e.*, at a temperature now well above *T_cp_*. We kept strictly the same microwave pumping power (475 mW) for both experiments whereas the X-ray photon energy (*E*_1_ *≃* 7114 eV) was left unchanged. The phase-shifts (ΔΦ) were determined according to the same criteria: the dispersive part of XDMR spectrum had to pass through zero at resonance whereas the absorptive part had to be positive over the whole resonance spectral range. Whereas ΔΦ varies from +15*°* to −177*°*, it immediately appears that something unexpected did happen to the absorptive part which caused heavy distortions of the modulus (*|*XDMR*|*) and of the PSD spectra. In contrast, no anomaly did appear in the FMR PSD spectrum which was recorded simultaneously at *T* = 450 K.

For sure, the strong anomaly revealed by Figure 6B cannot be explained by a simple inversion in the precession helicity of *M*^(^*^ℓ^*^)^ at the 
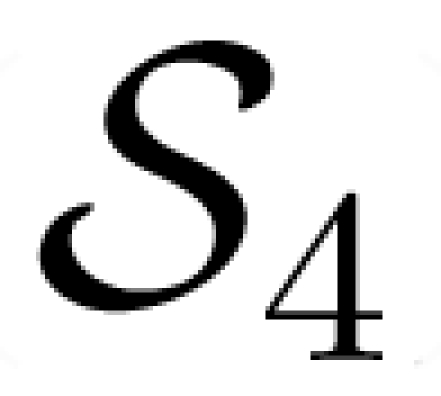
 iron sites (24d). It is our interpretation that this anomaly is typical of a destructive interference between two resonant modes precessing in opposite senses as illustrated (for example) with Figure 6C. In other terms, the polarization of the precession is getting strongly elliptical. Complementary experiments revealed that this anomaly strongly depended on the pumping power and decreased on increasing the temperature. This looks like a possible signature—in the transverse detection geometry—of a 4-magnon scattering process in which the two scattered magnons with wavevectors +**k** and −**k** had strictly opposite helicities. We suspect the destructive interference to be particularly spectacular because the linewidth of the non uniform modes (*±***k**) should be (again) much shorter than the linewidth of the degenerate uniform mode.

As far as the proposed interpretation holds true, the clue to the present problem is in the scattering amplitude 
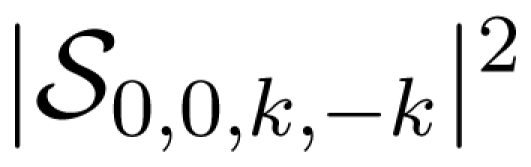
 which depends mostly on dipole-dipole interactions. It is not totally clear as yet why no similar anomaly did show up below the compensation temperature, *i.e.*, in Figure 6A. It is hard to anticipate what exact role the Gd spins play in the intermediate temperature range above the ordering temperature T*_B_* and below T*_cp_*. Note that ^57^Fe spin-echo NMR spectra recorded well below T*_B_* have established that the Gd spins were coupled to the Fe spins in the tetrahedral (24d) sites, the exchange integral *J_dc_* amounting to only *≃* 12% of the exchange integral *J_ad_* describing the strong ferrimagnetic coupling of the iron sites [70,71]. Further work is in progress in order to compare the saturation properties of low temperature XDMR spectra recorded at the Fe K-edge and at the Gd L-edges.

### 3.5. Non-Linear Effects Associated With Elliptical Precession

For iron garnets, the shape anisotropy is very often regarded as the main cause for elliptical precession in FMR: this is typically the case of YIG thin films when tangentially magnetized. *Oblique* magnetization at 45*°* or near the magic angle (54.74*°*) is a simple way to decrease the anisotropy fields and to minimize the foldover lineshape distortions at high pumping power. Nevertheless, the two experiments reported below unambiguously show that the precession still remains elliptical. It should be kept in mind, however, that the precession ellipicity is a time-reversal even observable property [7]: this implies that it can be measured only through very weak *non-linear* effects.

#### 3.5.1. Second Harmonic XDMR Spectra in YIG Thin Films

Under the conditions of elliptical precession, the longitudinal component of the magnetization *m_z_* is not anymore time-invariant since there is a weak additional term that is oscillating at twice the microwave pumping frequency and which appears as a consequence of the nonlinear character of the Landau– Lifshitz–Gilbert or Bloch–Bloembergen torque Equations [20]:

(42) $$m_{z}=m_{z}^{\left(0\right)}+m_{z}^{\left(2\right)}\;\text{cos}(2\times \omega_{p}t+\varphi^{\left(2\right)})$$

Clearly, the detection of such a weak frequency-doubled XDMR signal was a critical test regarding the sensitivity of the superheterodyne detection scheme. It is noteworthy that some phase information can be recovered using a vector detection of the frequency doubled signal. It can be shown that *m_z_* ^(2)^ as well as *m_z_*^(0)^ are time-reversal odd observables that can perfectly be detected by XMCD as long as the oscillating term preserves its time-even parity. This will obviously be the case if the precession helicity is inverted in order to keep 2*ωt* invariant.

**Figure 7:**
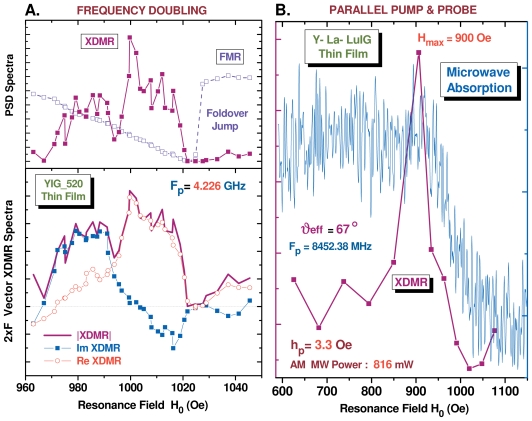
(**A**) Vector Components and PSD XDMR spectra of the YIG film **1** recorded in the frequency doubling mode and longitudinal detection geometry (LOD); the pumping frequency was 4.226 GHz but the XDMR signal was oscillating at 8.452 GHz. Note that the 2 *× F* XDMR PSD spectrum does not reproduce the foldover lineshape of the FMR PSD spectrum. Regarding the vector 2 *× F* XDMR spectra, it seems that resonance may also take place at lower field; (**B**) Parallel pumping PSD XDMR spectrum of the Y-La-LuIG film **2** recorded in LOD geometry under high pumping power at 8.452 GHz. Whereas the microwave absorption spectrum due to the parametric excitation of exchange spin waves extends down to very low fields, this is not the case for the XDMR spectrum which exhibits only a sharp peak at *H_max_*.

The 2 *× F* vector spectra of film **1** which are reproduced in Figure 7A were recorded in pumping the sample in the microwave C-band (*F_p_* = 4.226 GHz) and in detecting the weak XDMR signal at 2 *× F* = 8.452 GHz using the 24th harmonics of the RF signal of the storage ring as local oscillator reference. Superheterodyne detection turned out to be possible using a single side-band frequency translator as modulator.

With a pumping power as high as 1.25 W, the foldover lineshape of the FMR PSD spectrum was not unexpected. Rotating the film at the magic angle (*θ_H_* *≃* 54.74*°*), however, considerably reduced the foldover linewidth (35 *<* Δ*H <* 50 Oe). Interestingly, the weak 2 *×* F XDMR PSD spectrum displayed in Figure 7A does not reproduce the foldover lineshape of the FMR PSD spectrum; moreover, it looks like it was peaking at a resonance field much lower than the foldover jump and closer to the resonance field found in FMR experiments carried at low pumping power. Actually, the 2 *× F* XDMR spectra shown in Figure 7A should not be compared with the PSD FMR spectrum but rather with the vector-type *excitation* spectrum obtained in measuring the radiated microwave intensity at frequency 2 *× F_p_*. To the best of our knowledge, no such FMR excitation spectra had ever been reported. Thanks to the recent upgrade of our instrumentation, we should now be able to record simultaneously the 2 *× F* vector XDMR and *excitation* FMR spectra and to compare them.

#### 3.5.2. XDMR under Parallel Pumping Excitation

We have reproduced in Figure 7B the XDMR PSD spectrum of film **2** recorded in the parallel pump and probe geometry (**b***_p_* *||* **B**_0_ *||* **k***_RX_*) illustrated with Figure 3C. In this geometry, some microwave power is absorbed due to the direct excitation of a pair of spin waves with opposite helicities +**k** and −**k** as predicted by Equation 20. Some more complication arose from the *oblique* pumping geometry with respect to the film. This is because the equilibrium magnetization **M***_eq_* is not aligned anymore along the direction of the external bias field *B*_0_ but along the direction of some *effective* field **B***_eff_*. A careful analysis of the angular dependence of the FMR spectra made it possible to determine the polar angle of **M***_eq_* in the film coordinates: *θ_eq_* *≃* 67 if *θ_H_*__0__ = 54.74*°*. Thus, in addition to a large component (*b||_p_*) parallel to **B***_eff_*, the microwave pump field **b***_p_* contributes to a weak transverse component (*b*_⊥*_p_*_) that simultaneously excites the uniform precession mode at frequency *ω_p_*. This explains why a standard FMR signal was systematically detected in this geometry [73].

We were primarily interested in the excitation of a pair of spin waves (*±***k**) by the large component *b||_p_*. Equation 20 predicts a maximum intensity for spin waves propagating along a direction perpendicular to **B***_eff_*. In the film coordinates, the wavevectors *±***k** have a component along the normal to the film (*±k_σ_*) as well as a component in-plane (*±k_π_*). In an infinite thin film, the *π*-components refer to progressive waves, whereas resonant standing waves develop along the normal to the film. In favorable cases, e.g., for high quality YIG films, one may detect a rich pattern of discrete standing wave resonances at pumping levels that exceed the threshold field defined by Equation 38 of Section 2.5. Unfortunately, this was not the case for the Y-La-LuIG film **2** for which we observed at low resonance field only a fairly broad absorption band that is reproduced in Figure 7B. It is quite noteworthy that the XDMR PSD spectrum recorded in longitudinal detection geometry (LOD) does not reproduce such a broad band commonly assigned to exchange spin wave resonances: there is a significant signal only at the onset of what is given as the exchange magnon band, *i.e.*, for *k ≃* 0. In other terms, this experiment seems to indicate that, at the iron sites, the orbital magnetization component *M*^(^*^ℓ^*^)^ measured at the Fe K-edge *does not* couple to exchange spin waves, at least to those featuring a large wavevector *k*. Further work should tell us how far this conclusion can be granted as general, and whether there is any link between this observation and the well known property of the exchange Hamiltonian to commute with either M*_z_* or m*_z_*.

## 4. New Challenges for XDMR

So-far, we discussed XDMR spectra recorded only on ferrimagnetic films or crystals. In collaboration with the research center for the development of the far infrared region (University of Fukui, Japan), we are presently testing at the ESRF the feasibility of high-field XDMR experiments at sub-THz pumping frequencies [74,75]. Our ultimate goal is to evaluate whether high-field X-ray detected Electron Paramagnetic Resonance (EPR) spectra could be successfully recorded in longitudinal detection geometry (LOD). On the other hand, it will be shown below that high-field XDMR could also become a unique tool to study orbital magnetism in AFM phases.

### 4.1. High-Field, X-Ray Detected Electron Paramagnetic Resonance

**Figure 8:**
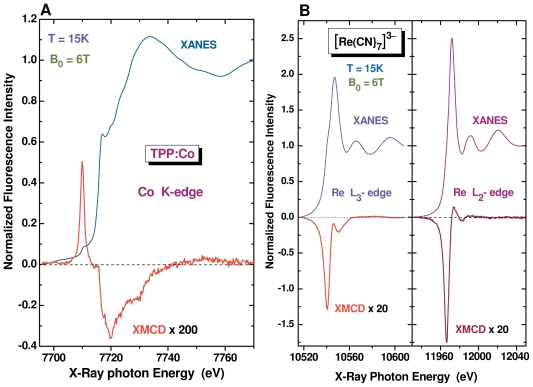
(**A**) XANES and XMCD spectra recorded at the Co K-edge for a powdered pellet of **3** = TPP:Co; (**B**) XANES and XMCD spectra recorded at the Re L_2_*_,_*_3_-edges for a powdered pellet of **4** = [Re(CN)_7_](Bu_4_N)_3_. Note that the XMCD signal measured at the Re L-edges is much stronger than at the Co K-edge.

At the ESRF, quite a few proposals were concerned with static XMCD studies on paramagnetic organometallic complexes. In practice, such experiments require low temperatures (*T ≤* 20 K) and a high magnetic field (*B*_0_ *≥* 5 T). We have reproduced in Figure 8A the cobalt K-edge X-ray Absorption Near Edge (XANES) and XMCD spectra of a powdered pellet of the meso-(5,10,15,20)-tetraphenylporphyrinato-Co(II) complex **3** = TPP:Co; we have also reproduced in Figure 8B the rhenium L-edges XANES and XMCD spectra of a cyanometalate complex of Re(IV) (**4** = [Re(CN)_7_](Bu_4_N)_3_). Recall that **4** is a building block for the chemical synthesis of heterobinuclear molecular magnets [76] or chemically switchable molecular magnet [77].

Interestingly, the XMCD spectra of complexes **3** and **4** are dominated by a strong contribution of the orbital magnetization component 〈*ℓ_z_*〉 in the X-ray excited final states. Since the integral of the XMCD signal reproduced in Figure 8A does not average out to zero, the magneto-optical sum rules let us anticipate the presence of a significant amount of unquenched orbital moment 〈*L_z_*〉 located at the cobalt sites. This is consistent with the commonly admitted electronic structure of **3** in which there is a close lying (orbital) triplet ^4^*T*_1_ slightly above the Kramer’s ground state ^4^*A*_2_. The existence of a weak orbital moment is also supported by the rather large anisotropy of the EPR spectra: Δ*g*^2^ = *g*_||_^2^− *g*_||_^2^*≃* −7.8 [78]. This is at variance with the case of vanadyl porphyrin complexes since the XMCD signals measured at the V K-edge were dramatically weak and close to the instrumental detection limit although the corresponding XANES spectra did show quite strong pre-edge structures [79]. This may look puzzling given that the EPR spectra of vanadyl complexes are known to be very intense and exhibit fairly narrow lines with well resolved hyperfine structures due to the ^51^*V*_7_*_/_*_2_ nuclei. Actually, the very weak anisotropy of the EPR spectra (Δ*g*^2^ *≃* −0.098) provides us with a clear indication that the orbital moment are heavily quenched in these compounds. Moreover, the weakness of the XMCD signal at the V K-edge could also reflect a major delocalization of the unpaired electron far away from the vanadium site.

It immediately appears from Figure 8B that the XMCD signatures measurement at the Re L_2_*_,_*_3_ edges are considerably more intense than in the previous case. Moreover, the XMCD spectra displayed in Figure 8B are fairly unusual since the XMCD signal keeps the same sign at the L_2_*_,_*_3_-edges: according to the magneto-optical sum rules, this is a typical signature of a large orbital moment 〈*L_z_*〉 = 0.11 *μ_B_*. This is also fully consistent with the strong anisotropy of the EPR spectra (Δ*g*^2^ *≃* 11) [76]. Much more surprising was the very weak effective spin contribution with 〈*S_z_*〉 − 7*/*2〈*T_z_*〉 *≃ ±*5.10^−4^ *μ_B_*. Quite the opposite situation was found when we measured the gadolinium L-edges XMCD spectra of cofacial porphyrinato-Gd(III) complexes in which no significant orbital moment was detected [79].

Unfortunately, the XDMR spectra of the paramagnetic complexes **3** and **4** *cannot* be recorded in the microwave X-band because the sensitivity of the XMCD probe is very poor at low bias field. A better option would be to record high-field XDMR spectra pumped at sub-THz frequencies. Even under such conditions, the Co K-edge XDMR signal may still be very weak. Nevertheless, one should not regard such a challenging experiment as hopeless because much larger precession cone angles could *a priori* be achieved in EPR: recall that pulsed EPR spectrometers require the magnetization to be rotated by 90*°* or even 180*°* in the rotating frame [80–83]. There is, unfortunately, a major difference with standard EPR which is that XDMR spectra will have to be recorded on pure or highly concentrated samples. Under such conditions, the X-ray detected electron paramagnetic resonance lines are expected to be very broad because the exchange narrowing effect should be considerably weaker than in FMR [84,85]. From a technical point of view, there is also the further handicap that the superheterodyne detection scheme discussed in Section 2.6 cannot be extended beyond *F_p_* = 20 GHz: this implies that, as yet, X-ray detected EPR spectra could be recorded at sub-THz frequencies only in the longitudinal detection geometry (LOD) which suffers from a very poor sensitivity. This point justifies the need for a powerful pumping source such as a gyrotron [74,75].

What stimulates us to invest time and efforts in this challenging project is the hope that high-field X-ray detected EPR experiments should allow us to probe the precession dynamics of orbital magnetization components in paramagnetic species with a significant zero-field splitting (zfs), e.g., high spin complexes with integer spin that are EPR-silent at microwave pumping frequencies. Many examples can be found in a long list of Mn(III) complexes [86], e.g., porphyrinato-Mn(III) complexes for which preliminary XMCD measurements at the Mn K-edge already confirmed the existence of unquenched orbital moments. Keeping in mind that zfs is caused by spin-orbit interactions, there should be a correlation between a large zfs and the magnitude of M^(^*^ℓ^*^)^ in the X-ray excited final states. In other terms, spin-orbit is responsible for both the large zfs of Re(IV) complexes and the large contribution of 〈*L_z_*〉 and 〈*ℓ_z_*〉 in XMCD. Complexes involving 5*d* transition metal elements would thus appear as excellent candidates to demonstrate the precession of orbital components in X-ray detected EPR.

The intriguing case of the Van Vleck paramagnetism still deserves a few comments here: it can be best observed when the angular momentum *J* vanishes whereas *L, S* ≠ 0. If the ground state is singlet, the first order perturbation due to the Zeeman interaction vanishes but the second order term yields the Van Vleck susceptibility that becomes temperature independent at low temperatures (*T <* 20 K):

(43) $$\chi_{VV}=2\mu_{B}^{2}(N/V)\sum_{n\neq 0}\frac{\langle 0|L_{z}+{gS}_{z}|n\rangle \langle n|L_{z}+{gS}_{z}|0\rangle }{E_{n}-E_{0}}$$

Whereas Curie’s paramagnetism reflects the alignment of *permanent* moments, Van Vleck’s paramagnetism refers to an electronic polarizability associated with *induced* moments. So far, van Vleck’s paramagnetism was observed mostly in crystals containing non-Kramers RE ions that (again) have an integer spin and a large zfs. What is required from the crystal field is to split the atomic ground multiplet so as to produce a singlet ground state. A typical example is Eu^3+^ with its ground state ^7^*F*_0_ separated from the first excited state by *ca.* 300–400 cm^−1^ and for which a slow longitudinal relaxation process was proposed by Van Vleck [67]. In NMR, there is also much interest in the Van Vleck paramagnetism of thulium crystals which had an exceptional potentiality regarding dynamic nuclear polarization (DNP-NMR) of ^169^Tm. One of the best characterized crystals is thulium ethylsulphate, *i.e.*, TmES = Tm(C_2_H_5_SO_4_)_3_.9H_2_0 for which high-field EPR spectra have been reported at 1.2 K [87]. For the latter experiments, TmES was diluted in a diamagnetic crystal (LaES), the pumping frequency being as high as 1–1.5 THz. Unfortunately, we have not yet the technical capability to manage XDMR experiments under high pumping power at 1 THz while maintaining the sample temperature below 10 K.

### 4.2. High-Field XDMR in the AFMR Regime

**Figure 9:**
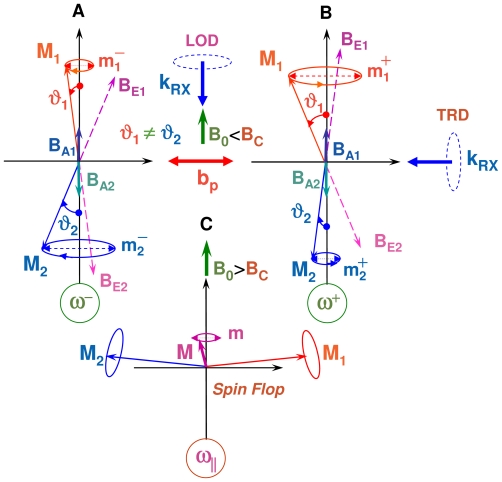
Precession modes in an antiferromagnet with an easy axis of anisotropy: (**A**) and (**B**) refer to an antiparallel ground state; (**C**) is for a noncollinear ground state (spin-flop mode). Since the precession cone angles *θ*_1_ and *θ*_2_ are unequal in configurations (**A**) and (**B**), a weak XDMR signal may still be detectable in the transverse detection geometry (TRD).

Since no static XMCD signal can be measured on AFM materials with antiparallel ground states, it is tempting to conclude that there is no hope to detect any XDMR signal as well. The situation may not be as desperate if one looks at what happens under the conditions of antiferromagnetic resonance (AFMR). For simplicity, let us consider a crystal with two AFM ordered sublattices and uniaxial magnetic anisotropy directed along the **c** axis. As long as the antiparallel ground state is preserved, the combined action of a parallel bias field (*B*_0_ *||* **c**) and a perpendicular pumping field (*b_p_* ⊥ **c**) will excite two precession modes illustrated with Figures 9A,B and which satisfy the resonance condition:

(44) $$\frac{\omega^{\pm }}{\gamma }≃{\left[2B_{E}\cdot B_{A}+B_{A}^{2}\right]}^{1/2}\pm B_{0}=B_{C_{2}}\pm B_{0}$$

in which *B_E_* = *|***B***_E_*_1_ *|* = *|***B***_E_*_2_ *|* is the modulus of the exchange fields of the coupled sublattices (1*,* 2), whereas *B_A_* = *|***B***_A_*_1_ *|* = *|***B***_A_*_2_ *|* similarly refers to the length of the corresponding anisotropy fields. Recall that Equation 44 is correct only at low temperatures (*T ≪* T*_N_*) and neglects all nonlinear terms. Further corrections taking into account the Van Vleck paramagnetism are possible when the temperature dependent susceptibility ratio *χ_||_/χ_⊥_* is predetermined [88].

Anyhow, two distinct precession modes (*ω*^+^; *ω* ^−^) are excited which have the opposite helicity. Moreover, due to the existence of the anisotropy field (**B***_A_* = **B***_A_*_1_*_,_*_2_ ), the precession cone angles *θ*_1_ and *θ*_2_ are no longer identical for the two magnetization components *M*_1_ and *M*_2_. In the transverse detection geometry (TRD) illustrated with Figure 9, one should then measure a *difference* XDMR signal proportional to Δ*m_⊥_^±^*= *m*_1_*^±^*+ *m*_2_*^±^* and which should oscillate at the resonant frequencies *ω*^+^ or *ω* ^−^ with the following lineshape [20]:

(45) $$\Delta m_{⊥}^{\pm }=m_{1}^{\pm }+m_{2}^{\pm }≃\frac{2\gamma^{2}B_{A}}{(\omega^{+}-\omega )(\omega^{-}-\omega )+21\alpha_{G}\omega B_{E}}b_{cp}^{\left(\pm \right)}$$

in which *b_cp_*^(^*^±^*^)^ is the relevant circularly polarized component of the pump field, *α_G_* being again a dimensionless (Gilbert) damping parameter. There is, however, the considerable handicap that the zero-field resonance frequency, *i.e.*, *ω_C_* = *γB_C_*__2__ is most often expected to be in the sub-THz, if not in the far-infrared range because the exchange fields (**B***_E_*__1__, **B***_E_*__2__) are much stronger than the external bias field. Recall that there is, as yet, no X-ray detector that can measure a signal oscillating at sub-THz frequencies, our superheterodyne detection scheme being restricted to *F_p_* *≤* 20 GHz. This is where high-field XDMR could circumvent this difficulty, assuming that the external field *B*_0_ can be strong enough to shift the resonance frequency *ω* ^−^ down to the microwave range. Notice that, at the ESRF, the static bias field applied at the sample location could now be increased up to 17 T.

When the external field *B*_0_ approaches the critical field *B_C_*__1__ = [2*B_E_B_A_* − *B_A_*^2^ ]^1^*^/^*^2^ *< B_C_*__2__, the magnetic system undergoes a first order transition (spin-flop) resulting in the new situation sketched in Figure 9C and which is characterized by a noncollinear ground state [88]. It is well documented that, in the spin-flop phase, a new resonance mode associated with the total magnetization *M* = *M*_1_ +*M*_2_ will be pumped:

(46) $$\frac{\omega_{\left|\right|}}{\gamma }≃{\left[\frac{2B_{E}B_{E⊥}}{B_{\left|\right|}^{2}}B_{0}^{2}-2B_{E}\cdot B_{A}\right]}^{1/2}$$

in which: *B_E||_*= 2*B_E_* − *B_A_* and *B_E⊥_*= 2*B_E_* + *B_A_*, so-that *B_C_*__1__ = [*B_A_B_E||_*]^1^*^/^*^2^ and *B_C_*__2__ = [*B_A_B_E_*_⊥_]^1^*^/^*^2^. For completeness, it should be mentioned that there exists also a *soft* mode (*ω →* 0) but the latter will be undetectable by XDMR since it cannot be excited by the external pump field [20].

Given that the precession cone angles *θ*_1_ and *θ*_2_ are different, one may question whether a (very weak) XDMR signal could be measured as well in the longitudinal detection geometry (LOD), *i.e.*, in a regime where non-linear terms would no longer be neglected. Since no fast detector is needed in LOD geometry, this option could still be envisaged when *B_C_*__2__ is very large, *i.e.*, when too large magnetic fields would be required to shift *ω* ^−^ down into the microwave X-band. Unfortunately, one expects the XDMR signal measured in LOD geometry to be much smaller than in FMR because Δ*m_z_^±^* *∝* [tan *θ*_0_]^2^ *×* (*B_C_/B_E_*)^2^, in which *B_C_/B_E_* does not exceed, at best, a few percents. This would bring us very close to (or below) the detection limit.

For XDMR experiments, it may be preferable to select AFM crystals in which the exchange field *B_E_* is not too strong, *i.e* crystals with a rather low Néel temperature (e.g., *T_N_* *<* 77 K). Regarding AFMR, MnF_2_ (*T_N_* = 68 K) is a good example given that a very narrow linewidth (Δ*B* = 5 G) was measured at *T* = 4 K for the uniform precession mode (*ω* ^−^) excited by a microwave pump field (F*_p_* = 23 GHz) in a bias field *B*_0_ *≃* 8.5 T [89] so that magnetostatic satellite resonances could be perfectly resolved. Narrow AFMR lines (Δ*B* = 15 G) were also obtained with epitaxial films grown on MgF_2_ substrates [90]. Note that the AFMR linewidth rapidly increases in the sub-THz range since the damping term in Equation 45 is *∝ ωB_E_*. Unfortunately, MnF_2_ may not be the ideal candidate for Mn K-edge XDMR experiments due to the vanishing orbital moment 〈*L_z_*》 of Mn^2+^ ions in a high spin (^6^*S*) state. Recall, however, that 〈*ℓ_z_*〉 may still be finite in excited states even though 〈*L_z_*〉 vanishes in the ground state. There is the further handicap that, in an octahedral crystal field with ^6^*A*_1_*_g_* ground term, only electric quadrupole (*E*2) transitions contribute to the pre-edge structures assigned to final states involving magnetic 3d orbitals.

The case of KCuF_3_ (*T_N_* = 39 K) looks more attractive owing to the fact that this crystal is the archetype of orbitally ordered systems [91]. In this crystal, the AFM spin order has an easy plane of anisotropy with its normal parallel to the **c** axis. On the other hand, careful studies of the angular dependence of the EPR spectra of KCuF_3_ in the paramagnetic phase revealed the existence of a strong contribution of the Dzyaloshinskii–Moriya antisymmetric exchange interaction:

(47) $$\sum_{k>j}d_{jk}\cdot (S_{j}\times S_{k})$$

with **d***_jk_* ⊥ **c** [92]. Moriya established that such an antisymmetric exchange interaction could be identified with a second order perturbation which was bilinear in the spin-orbit coupling and exchange interaction [93]. Then, the coupling field **d***_jk_* could be expressed in terms of transition orbital moments:

(48) $$d_{jk}≃\frac{\lambda \cdot J_{j}}{\Delta E_{j}}\langle g_{j}|L_{j}|e_{j}\rangle -\frac{\lambda \cdot J_{k}}{\Delta E_{k}}\langle g_{k}|L_{k}|e_{k}\rangle$$

in which *λ* denotes the spin-orbit coupling factor, Δ*E_j_*_(_*_k_*_)_ are the energy separations between the ground state orbital levels *g_j_*_(_*_k_*_)_ and the relevant excited states *e_j_*_(_*_k_*_)_, *J_j_*_(_*_k_*_)_ being the effective superexchange constants at sites *j* and *k* respectively. Clearly, the coupling field vanishes if the two sites transform into each other by inversion symmetry. It is well documented that a large antisymmetric exchange interaction can cause a small canting effect of the AFM-ordered magnetization vectors resulting in a weak ferromagnetism [39]. Typically, for *B*_0_ ⊥ **c**, KCuF_3_ should behave just like a *weak* ferromagnet satisfying the resonance condition [20]:

(49) $$\omega_{⊥}=\gamma B_{0}{\left[1+B_{A}/B_{E}\right]}^{1/2}$$

Under such conditions, one may reasonably expect a (weak) XDMR signal to be detectable at low temperature in TRD geometry.

Among transition metal oxides for which the low frequency mode *ω* ^−^ could be excited at microwave frequencies, single crystals of ilmenites ( 
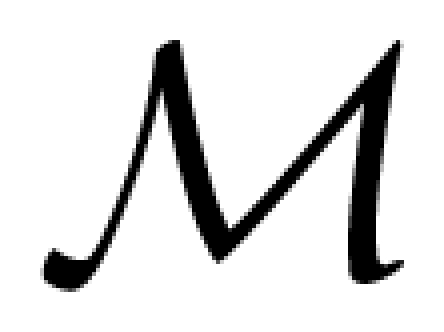
TiO_3_, with 
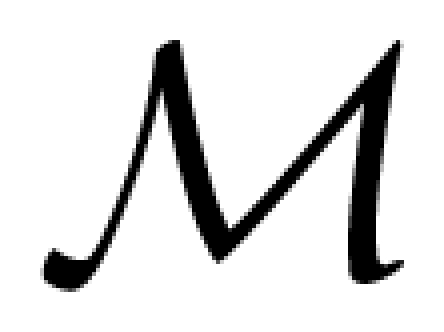
 = Fe,Co) could also be good candidates for XDMR: they had rather low Néel temperatures [88,94] and significant orbital moments 〈*L_z_*〉 at the sites of the Jahn–Teller cations [95]. With a much higher exchange field combined to a low anisotropy field [88,96], the case of Cr_2_O_3_ (T*_N_* = 308 K) looks less favorable even though we already produced definitive evidence of orbital magnetism in this crystal, at least in its magnetoelectric phase [97]. Although the spin-flop critical field of Cr_2_O_3_ is rather low (*B_C_*__1__ *≃* 5.9 T), it cannot be taken yet for granted that Δ*m*_⊥_^(^*^±^*^)^will be large enough to yield a XDMR signal easily detectable at microwave pumping frequencies.

Still very little is known regarding AFMR in crystals involving 5*d* transition elements and for which much stronger XDMR signals could be expected at L-edges. Unfortunately, neither single crystals nor epitaxial films of K_2_ReBr_6_ (*T_N_* = 15 K [39]) or ReO_2_ are easily available.

## 5. Conclusions

XDMR is a novel spectroscopy which is still in an early stage of development often dominated by severe instrumentation problems. In this review, we tried to convince the reader that XDMR could develop as a unique tool to study dynamical aspects of orbital magnetism including collective excitations. For the first time, direct experimental evidence was produced of the forced, elliptical precession of orbital magnetization components *M*^(^*^ℓ^*^)^. There is no doubt left that locally, *M*^(^*^ℓ^*^)^can couple to magnetostatic spin waves through dipole-dipole interactions but there is no experimental result which would definitively prove that *M*^(^*^ℓ^*^)^could similarly couple to exchange spin-waves. On the other hand, we pointed out strong anomalies in the XDMR spectra which support our view that orbitons may contribute to new relaxation mechanisms. Several important questions were left aside, e.g., the question to know whether, locally, the spin and orbital magnetization components M^(^*^s^*^)^ and M^(^*^ℓ^*^)^do precess in or out of phase and possibly around different effective axes. Also the whole problem of the magnetoelastic waves resulting from the modulation of the spin-orbit interactions by the lattice phonons will be discussed elsewhere. Nevertheless, in Section 4, we tried to anticipate over the emergence of XDMR as a parallel method to study orbital magnetism in paramagnetic as well as in antiferromagnetic phases.
